# Shedding Light on Cellular Secrets: A Review of Advanced Optical Biosensing Techniques for Detecting Extracellular Vesicles with a Special Focus on Cancer Diagnosis

**DOI:** 10.1021/acsabm.4c00782

**Published:** 2024-08-23

**Authors:** Beyza Nur Küçük, Eylul Gulsen Yilmaz, Yusuf Aslan, Özgecan Erdem, Fatih Inci

**Affiliations:** † UNAM—National Nanotechnology Research Center, Bilkent University, 06800 Ankara, Turkey; ‡ Institute of Materials Science and Nanotechnology, Bilkent University, 06800 Ankara, Turkey

**Keywords:** optical biosensors, extracellular vesicle (EV), EV isolation methods, EV biomarkers, cancer diagnosis

## Abstract

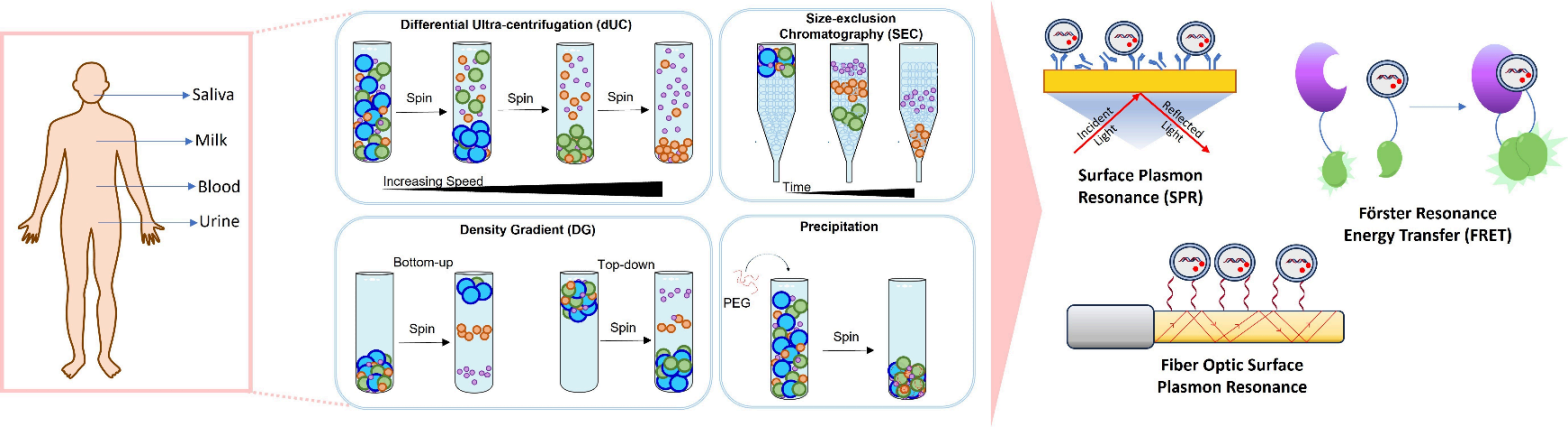

In the relentless pursuit of innovative diagnostic tools for cancer, this review illuminates the cutting-edge realm of extracellular vesicles (EVs) and their biomolecular cargo detection through advanced optical biosensing techniques with a primary emphasis on their significance in cancer diagnosis. From the sophisticated domain of nanomaterials to the precision of surface plasmon resonance, we herein examine the diverse universe of optical biosensors, emphasizing their specified applications in cancer diagnosis. Exploring and understanding the details of EVs, we present innovative applications of enhancing and blending signals, going beyond the limits to sharpen our ability to sense and distinguish with greater sensitivity and specificity. Our special focus on cancer diagnosis underscores the transformative potential of optical biosensors in early detection and personalized medicine. This review aims to help guide researchers, clinicians, and enthusiasts into the captivating domain where light meets cellular secrets, creating innovative opportunities in cancer diagnostics.

## 1 Introduction

**Figure 1:**
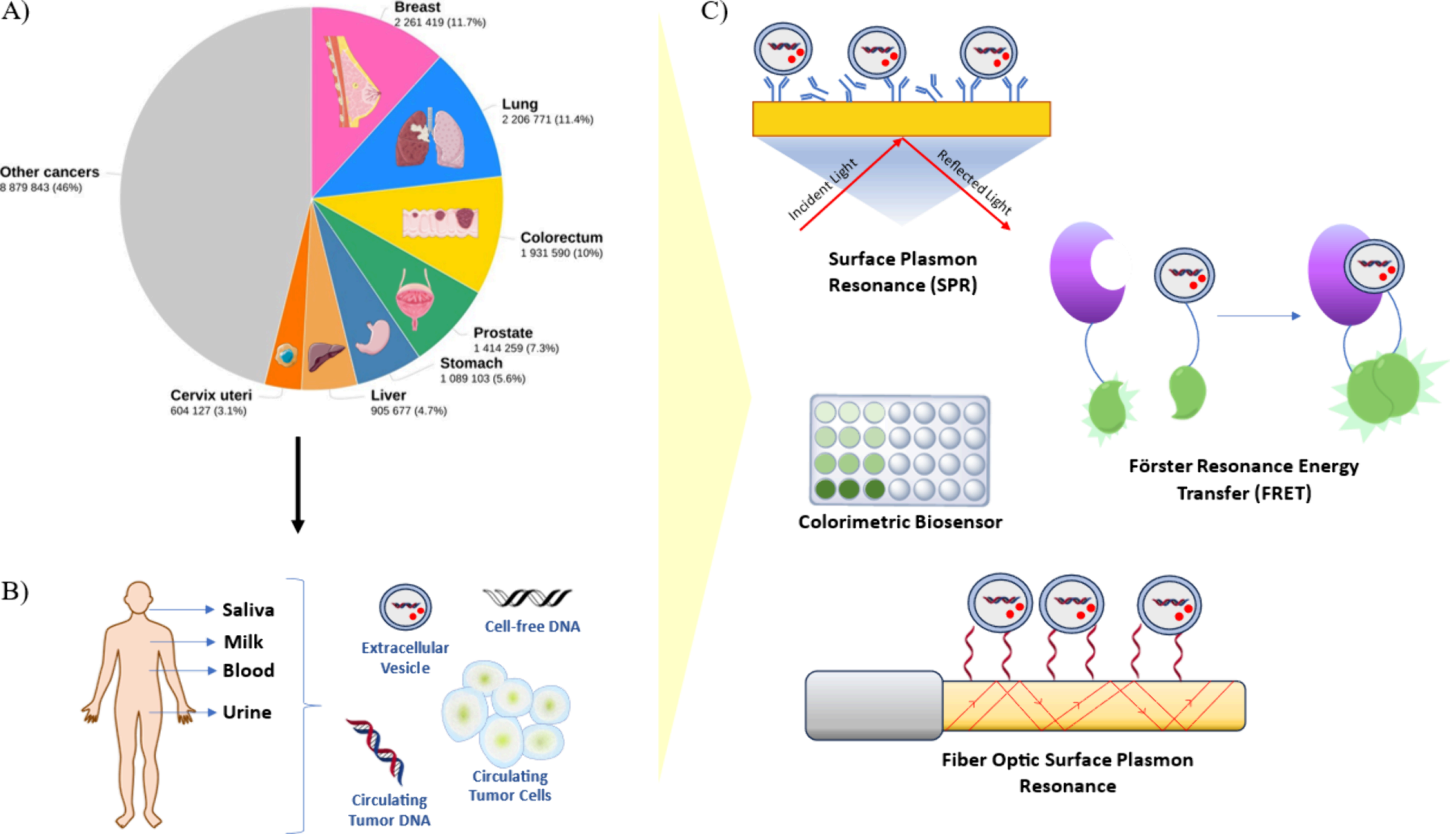
(A) A pie chart of the estimated number of cancer patients distributed around the world from all genders and ages is demonstrated. Retrieved from The Global Cancer Observatory (GCO). The figure is partly generated using Servier Medical Art, provided by Servier, licensed under a Creative Commons Attribution 3.0 unported license. (B) Biological fluids utilized in liquid biopsy and cancer biomarkers in them are illustrated. (C) Schematic of optical biosensors including SPR, FRET, FO-SPR, and colorimetric analysis for EV detection is demonstrated.

Cancer—a complicated set of illnesses that are defined by the uncontrolled proliferation and spread of abnormal cells to healthy tissues—has been the leading cause of deaths worldwide for decades (Figure 1A). The World Health Organization (WHO) declared that in 2020 nearly 10 million people died due to cancer. Detecting cancer at an earlier stage increases the likelihood of a favorable response to therapy, and thereby this potentially leads to a higher survival with a lower mortality rate, as well as reduced costs in clinical management including treatment processes. In practice, diagnostic methods based on physical examination such as computerized tomography (CT), magnetic resonance imaging (MRI), positron emission tomography (PET), ultrasound, and X-ray scans involve high radiation and pretreatment with lower sensitivity.^1,2^ To circumvent the challenges posed by conventional diagnostic methods such as invasive tissue biopsies and labor-intensive laboratory tests, the medical community has turned to liquid biopsy as an alternative approach. Liquid biopsy entails a minimally invasive method for analyzing cancer-derived biomarkers, such as miRNA,^3^ proteins,^4^ circulating tumor cells (CTCs),^5^ and extracellular vesicles (EVs) present in bodily fluids. This innovative technique offers the advantage of being less invasive and cost-effective and requiring less expertise, thereby representing a promising avenue for cancer diagnosis and monitoring (Figure 1B). Although EVs were initially regarded as cellular waste products, it is now widely acknowledged that they play a pivotal role in mediating communication between cells. EVs serve as carriers for cargo containing important biomolecules, including proteins, nucleic acids, and lipids. This recognition underscores the essential role of EVs in facilitating intercellular communication and their significance in various physiological and pathological processes.^6−8^ Moreover, given that EVs are reflective of their originating cells, they actively participate in both normal physiological processes and pathological conditions, including cancer progression and metastasis.^9,10^ While universal EV surface proteins such as CD9, CD63, and CD81 are widely recognized, cancer-derived EVs can harbor specific proteins indicative of their malignant origin. This specificity highlights the potential of EVs as diagnostic and prognostic markers in cancer research and underscores their role in mediating tumor-associated processes. Some of those include PD-L1, EpCAM, HER2, and MUC1.^11^ In spite of having great potential as a diagnostic tool, costly, time-consuming, intricate, and insufficient isolation and detection methods for EVs hamper their expansion in clinical use.

In the relentless quest for innovative diagnostic tools in the field of cancer research, optical biosensor systems were offered as an alternative solution for cancer-cell-originated EV detection since existing methods could be insufficient. Optical-biosensor-based diagnosis, such as surface plasmon resonance (SPR), surface-enhanced Raman spectroscopy (SERS), and colorimetric and fluorescence methods, exhibits superior features to conventional methods, including rapid response time and construction, immediate data acquisition, portability, and label-free detection (Figure 1C).^12,13^ Additionally, these methods demonstrate improved sensitivity and specificity to their targets through the decoration of antibodies and binders.^14,15^ Consequently, these biosensors have significant promise in the realm of cancer diagnostics, offering prospective applications across diverse cancer types.^16,17^

In this review, our primary objective is to underscore the significance of detecting EVs in the realm of cancer diagnosis through the utilization of optical sensors as a sensitive detection method. Herein, we initially present an overview of EVs, elucidating their origin and conventional detection methods. Subsequently, we expound upon optical sensors as an alternative strategy for EV detection and isolation. We then delineate the existing technologies and models currently available for this purpose. Finally, we discuss the pros and cons associated with optical-sensor-based detection of EVs, offering insights into the potential of this approach for enhancing cancer diagnostic methodologies.

## 2 Role and Importance of EVs in Cancer Diagnosis

To facilitate intercellular communication via the transfer of biological payloads, a wide array of mammalian cells, including pericytes, endothelial cells, and tumor-associated fibroblasts, actively secrete EVs.^18^ Their biological roles and functions, biogenesis, content, and biotechnological applications for therapeutic and theragnostic purposes have been extensively investigated and documented by various researchers.^19−21^ Notably, the International Society for Extracellular Vesicles (ISEV) has recently unveiled the most recent international guidelines for EV separation and characterization, termed MISEV 2023, with regular updates anticipated.^22^

**Figure 2:**
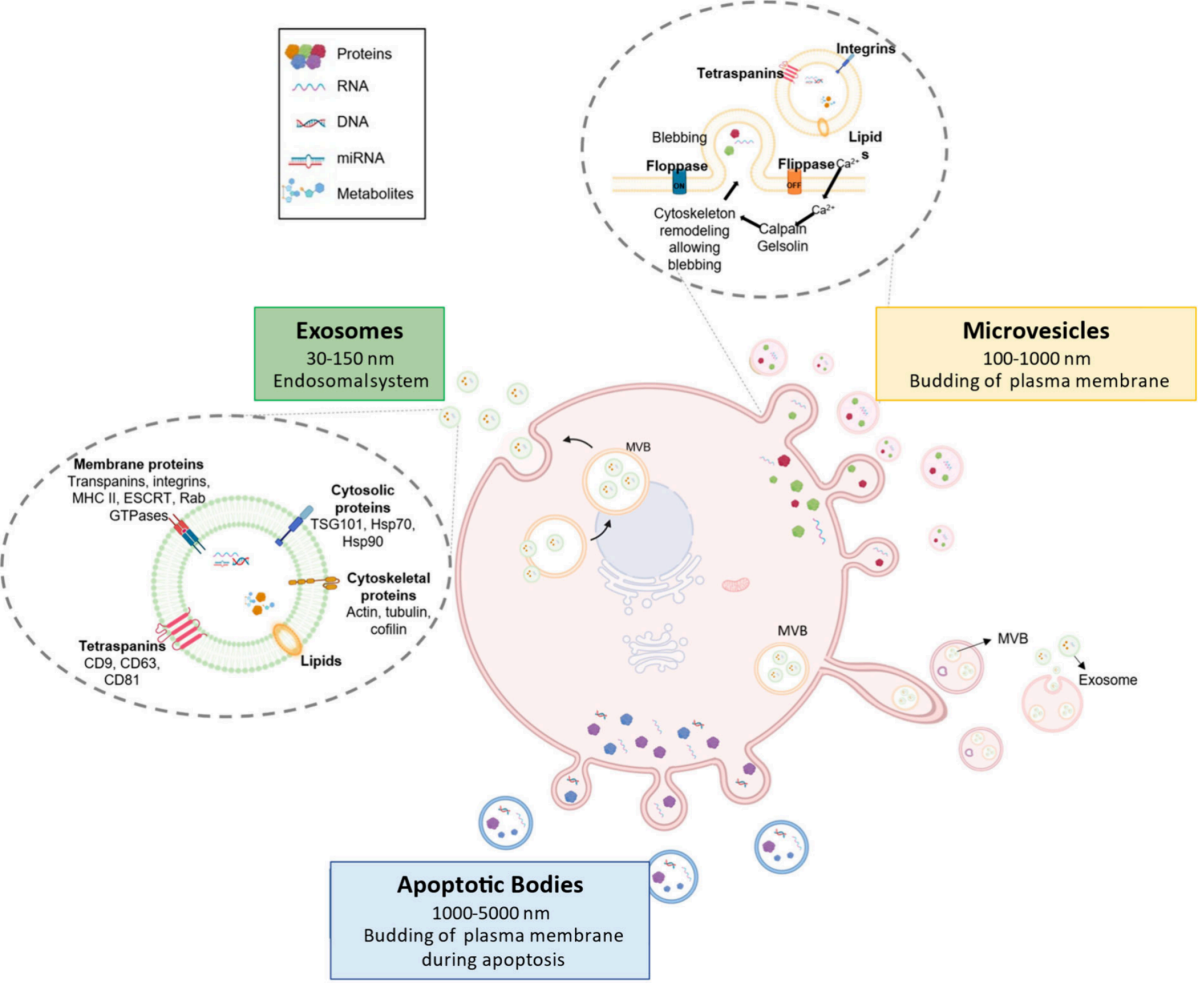
Origins and distinguishing features of EVs are shown. Exosomes, which have a size range of 30–150 nm, are formed through the process of inward budding from endosomal multivesicular bodies. Microvesicles, with sizes ranging from 100 to 1000 nm, are generated and released through the process of outward budding from the plasma membrane. ApoBDs (1000–5000 nm) are a distinct category of microvesicles that arise as a result of planned cell death within a cell. From ref (153). CC BY 4.0.

Advancements in cancer research have been accelerated by mounting evidence that EVs transport anticancer medicines like as microRNAs (miRNAs), nucleic acids, lipids, and specific proteins. The ability of EVs to convey information to recipient cells and modulate their function underscores their paramount significance. Given their diverse roles in physiological and pathological processes, such as immune response modulation and tissue homeostasis, EVs have recently garnered significant attention from researchers. Particularly in the context of cancer progression, EVs have emerged as pivotal regulators of the immune response, enlightening their role in shaping the tumor microenvironment and influencing disease outcomes.^23^ While chemokines, cytokines, and growth factors have long been thought to play a role in tumor cell–cell communication, EVs have recently come to light as new regulators of this process thanks to advancements in cancer therapy research. Briefly, EVs can be classified into three distinct categories considering their sizes (Table 1 and Figure 2). The first category consists of exosomes, which are formed within the endosomal network and are released when multivesicular bodies fuse with the plasma membrane.^24^ The second category includes microvesicles, which are generated by the plasma membrane through outward budding and fission.^25^ The latter, microvesicles (MVs), alternatively referred to as shedding vesicles or shedding microvesicles (SMVs), are small extracellular vesicles released by cells. Additionally, apoptotic bodies (ApoBDs) or apoptotic blebs are discharged as cellular blebs during the process of apoptosis.^26^ Exosomes are one of the types of EVs ranging from 30 to 150 nm in diameter.^27^ After multivesicular body–plasma membrane fusion, smaller vesicles are released. Intraluminal vesicles, also known as exosomes, are formed when multivesicular bodies form and are mostly present in all cell types and physiological fluids.^28^ Their contents fluctuate in viral infections, neurological illnesses (Alzheimer and Huntington diseases), and cancer; hence exosomes are extensively studied as biomarkers.^29^ Cellular origin also affects exosome protein and lipid content. Tetraspanins (CD63, CD81, CD82, and CD9), heat-shock proteins (Hsp60, Hsp70, and Hsp90), and annexins are the most prevalent exosomal proteins.^30^ Valadi et al. demonstrated exosome-mediated RNA transfer in 2007.^31^ Many studies have shown that exosomes transport mRNAs and miRNAs and that they receive and convey messages between cells.

**Table 1:** Characteristic Features of Extracellular Vesicles

	exosomes	microvesicles	apoptotic bodies	ref
size (nm)	30–150	100–1000	1000–5000	(24, 27, 32)
protein components	multivesicular bodies (TSG101, ALIX), tetraspanins (CD63, CD9, and CD81)	cell binding molecules (integrins and selectins), death receptors like CD40 ligand	transcription factors and histones	(156)
biogenesis	several multivesicular bodies join together in endosomal networks	through the process of plasma membrane budding	made by cells that are undergoing death	(157)
markers	tetraspanins (CD63, CD81, CD9) ALIX, flotillin, GTPases tetraspanins, HSP70, HSP90, TSG101, ESCRT, and MHC	metalloprotease surface phosphatidylserine, glycophorin, integrin (B1), selectin, MMP, flotillin-2, CD34, CD40, and CD45	annexin V, TSP, calnexin, surface phosphatidylserine histones, cytochrome *c*, histones, and C3b	(158−160)
lipid composition	low phosphatidylserine exposure, ceramide, lipid rafts, cholesterol, and sphingomyelin	high levels of phosphatidylserine and cholesterol	enriched in phosphatidylserine	(161, 162)
morphology	cup-shaped	cup-shaped	heterogeneous	(163−165)

Plasma membrane budding generates outer-layer MVs, and they are typically 100 nm–1 μm wide.^32^ The synthesis of MVs entails the involvement of various cellular components, including microtubules, actin filaments, and motor proteins such as kinesins and myosins, as well as SNAREs and anchoring proteins. However, the precise mechanistic details of this process remain elusive.^33^ MV production and utilization depend on donor and receiver cell microenvironments and physiological states. They may contain heat-shock proteins, cytoskeletal proteins, integrins, and post-transcriptional glycosylation and phosphorylation proteins.^34^ MVs, like other extracellular vesicles, help remove cellular waste. They can influence recipient cell response during intercellular communication, according to EV biology. Growth factors, hormones, and cytokines affect cellular activity and communication, whereas extracellular vesicles carry different biological contents. EVs carry proteins, nucleic acids, and miRNAs by unknown methods.^35^ Malignant cells can control their behavior by interacting with healthy cells.^36^ Cancer proteins are transmitted to healthy cells via EVs, encouraging metastasis. Understanding regulatory mechanisms and creating MVs may inspire new theragnostic methods.

Dying cells release vesicular ApoBDs within a size range of 1000–5000 nm.^24^ Depending on their size, structure, and composition, ApoBDs may be more prevalent than exosomes or MVs. The emerging recognition of EVs as carriers of genetic information within mammalian cells and organs underscores their increasing prominence. However, the relevance and potential roles of these enigmatic apoptotic entities necessitate further investigation. Despite extensive discussion in the literature, the cellular pathogenic processes and morphological alterations that govern their function remain largely unknown.^37^ It is widely acknowledged that ApoBDs contain substantial amounts of RNA compared to other EVs. Furthermore, larger ApoBDs may encapsulate DNA, RNA, lipids, and proteins.^38^ Consequently, ApoBDs possess the potential to influence downstream cells or recipients due to their extensive molecular reservoir.

### 2.1 Isolation Methods for EVs

**Figure 3:**
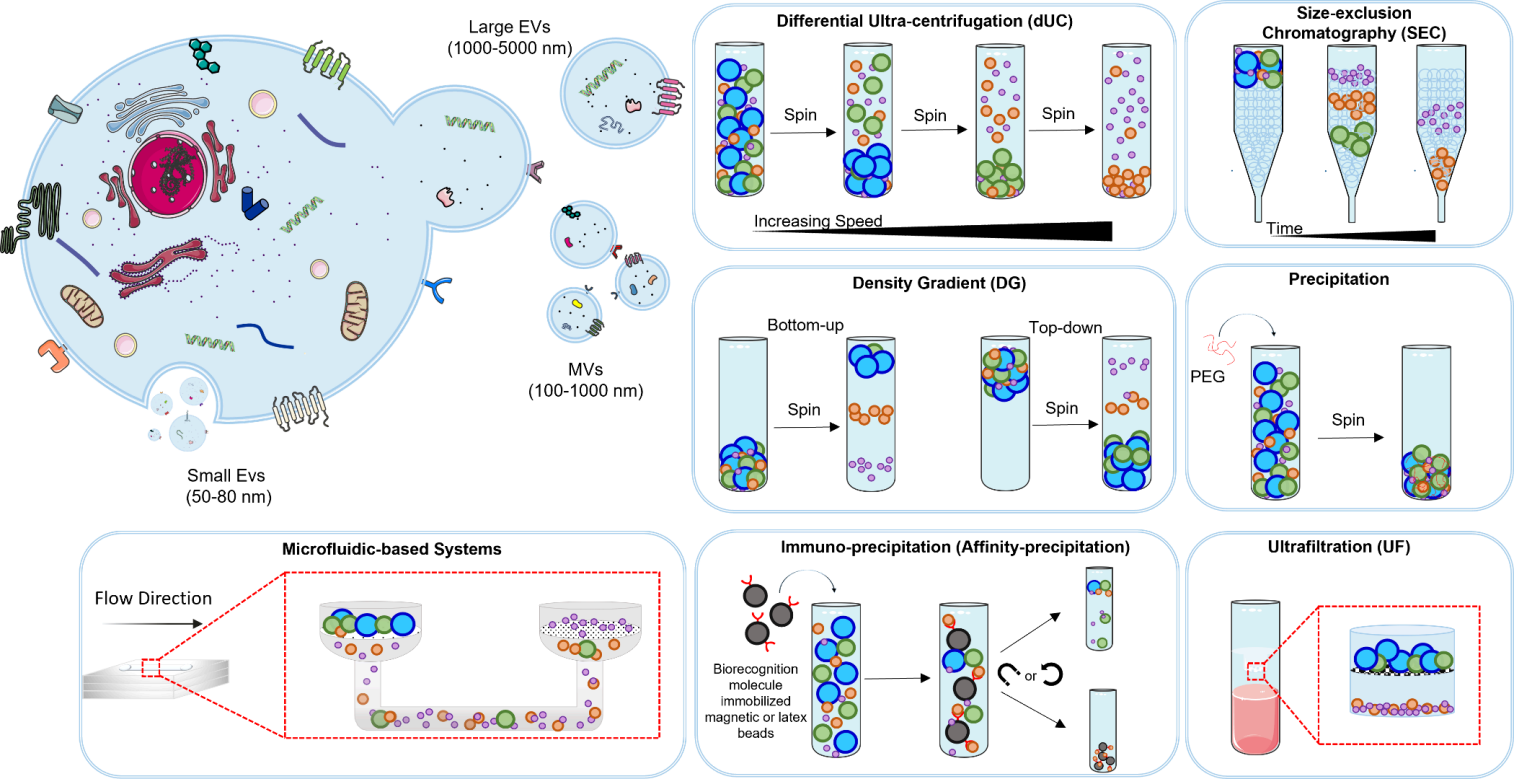
Methods used for EV isolation are shown. dUC and SEC are used to isolate EVs. To achieve differential elution of molecules with a speed–size inverse relationship, SEC employs biofluids as the mobile phase in opposition to a porous stationary phase. The elution process takes longer since larger particles elute first and smaller vesicles enter and flow through the pores second. EV subpopulations are separated by successively increased acceleration rates in dUC. There are also innovated methods for this regard. Solution-based PEG precipitation helps polymer-entrapped vesicles coalesce in huge numbers. EVs are efficiently captured by microfluidic (MF) chips with two filtered integrations. In immune-precipitation capture, antibodies against EV surface proteins isolate specific vesicles. UF uses a filter with a specified pore size to produce a vesicle-rich filtrate. From ref (41). CC BY 4.0. From ref (22). CC BY 4.0.

In the literature, a plethora of methods have been elucidated for isolating EVs from diverse biofluids, including cell culture media, milk, urine, and blood.^39,40^ While conventional methods like differential ultracentrifugation (dUC) are often considered the gold standard for EV isolation, a range of alternative techniques have emerged, each offering distinct advantages and limitations (Figure 3).^41^ These alternatives encompass ultrafiltration, precipitation agents such as polyethylene glycol (PEG), immunoaffinity capture, microfluidics, and size-exclusion chromatography (SEC). The conventional approach of dUC entails subjecting samples to a series of centrifugal forces and durations, thereby segregating particles based on their size and density, leading to their stratification into distinct sediment layers.^42^ Widely regarded as the gold standard method for isolating EVs, dUC is lauded for its ability to yield relatively pure populations of EVs, but in practice, pure isolation of EVs is not easily achievable. Pellets isolated using differential dUC typically exhibit minimal contamination from non-EV-related proteins; however, they may also precipitate lipoprotein or diverse particles with similar physical and biochemical properties. Moreover, applying a higher *g*-force for longer periods may cause EVs to aggregate. Centrifugation parameters must be optimized considering the biological fluid type (such as blood, urine, or milk) and interested EV subtypes (such as small EVs or large EVs) to prevent overlapped sedimentation areas. Another significant challenge associated with this technique is the considerable expense associated with its setup.^43^

Different from dUC, ultrafiltration relies on membranes with specific pore sizes to selectively filter particles based on size. In the literature, membranes with pore sizes of 0.8 and 0.45 μm are commonly employed to exclude larger particles, yielding an EV-rich filtrate.^44^ Conversely, membranes with pores smaller than the intended size of EVs (e.g., 0.22 and 0.1 μm) effectively remove smaller-sized particles.^45^ The range of EV sizes isolated through ultrafiltration is determined by the pore sizes of the membranes used, with the largest and smallest EVs retained by the first and last filtration membranes, respectively. This versatile method can be utilized to separate large microvesicles and exosomes either independently or in conjunction with ultracentrifugation. Even though this method is commonly preferable in terms of cost efficiency, membrane clogging and limited selectivity due to the size-dependent separation are disadvantages.^10,22^ Versatile usage of ultrafiltration with the combination of selectivity-based methods results in more accurate isolation.

In the PEG-based precipitation approach, EVs are initially encapsulated within a water-based PEG solution, facilitating the formation of aggregates that can be subsequently precipitated through low-speed centrifugation at 1500*g*.^46^ While the isolated EV size range remains consistent with that obtained through methods such as dUC, the specificity and purity of the precipitated EVs are compromised due to the coprecipitation of soluble non-EV proteins.^43^

Alternatively, the immunoaffinity capture approach involves the separation of EVs based on surface protein expression that results in more selective isolation for different EV subtypes. Key surface markers, such as CD9, CD63, and CD81, are frequently targeted using specific antibodies. To execute immunoaffinity capture of EVs, the sample material can be incubated with magnetic beads or ferric oxide nanocubes coated with antibodies against surface proteins, which are often conjugated with gold.^47,48^ One of the main drawbacks of immunoaffinity-based separation is that, if the antibody–antigen complex does not dissociate, the formation may interfere with further applications such as downstream analysis (Western blot or BCA). Moreover, target-specific antibody utilization is costly, and binding sites are limited.^10,22^

Size-exclusion chromatography (SEC) is another approach, and it relies on two main components: biofluids containing the target particles and porous gel filtration polymers. The stationary phase of SEC facilitates the elution of particles, vesicles, and proteins based on their size, with larger entities eluting first.^49^ This elution pattern occurs since larger particles have a shorter path to the column end and may pass through fewer pores, resulting in quicker elution compared to smaller particles. Common components of the stationary phase or chromatography column include Sephadex, agarose, Biogel P, and allyldextran.^41^ SEC is an accessible method with size-based separation without damaging the EVs; however, because there is a stationary phase, SEC separation may increase the sample volume and decrease the sample concentration compared to the initial sample.^22^ For pure EV isolation, more than one cycle may be needed with precise control of the elution process. In light of this, both pre- and post-SEC samples should be kept and analyzed in terms of concentration.

In contrast, microfluidic devices offer a high-throughput approach to isolating EV through immunoaffinity, size, and density based separation mechanisms.^50,51^ These devices leverage the principles of microfluidics to manipulate fluids and particles at the microscale, enabling precise control over isolation parameters and enhanced efficiency in EV isolation. Similar to immunoaffinity isolation, immuno-microfluidics has emerged as a prominent method for the isolation of EVs. This approach involves the use of specific antibodies immobilized on microfluidic chips to selectively bind to EV markers, facilitating their separation. For example, the CD63 antibody based ExoChip microfluidic technology has been employed for the isolation of EVs.^52^ Another noteworthy microfluidic technique involves a hybrid approach that integrates both dead-end and cross-flow processes within a single microfluidic chip.^50^ This innovative design combines the advantages of both approaches, potentially leading to a high recovery rate and reduced contamination. By leveraging the benefits of both dead-end and cross-flow filtration, this hybrid microfluidic platform offers enhanced efficiency and purity in EV isolation. Combining microfluidic strategies with size- or affinity-based approaches has a certain advantage to isolating EVs from small volumes, but bulk purification methods are more convenient for larger volumes. Microfluidic device fabrication requires specialized equipment, and they may vary from batch to batch. Their characterizations should be conducted in each usage.^13,22^

EVs are pervasive in numerous bodily fluids, spanning from blood and urine to peritoneal, lacrimal, synovial, cerebral, bronchoalveolar, and seminal fluids. These vesicles serve as carriers for a diverse array of substances, including lipids, polypeptides, membrane-bound and cytosolic proteins, various RNA species, and DNA. Facilitating intercellular communication, blood coagulation, immunomodulation, cell differentiation, detoxification, embryogenesis, endocrinology, neurological function, cancer progression, and tissue repair and regeneration, EVs play multifaceted roles in physiological processes. Their source, function, and cargo content are important parameters to decide the most suitable isolation method. The selection of an appropriate separation method should be guided by the specific properties of the EV sources as well as the desired yield and specificity of the EVs.

**Figure 4:**
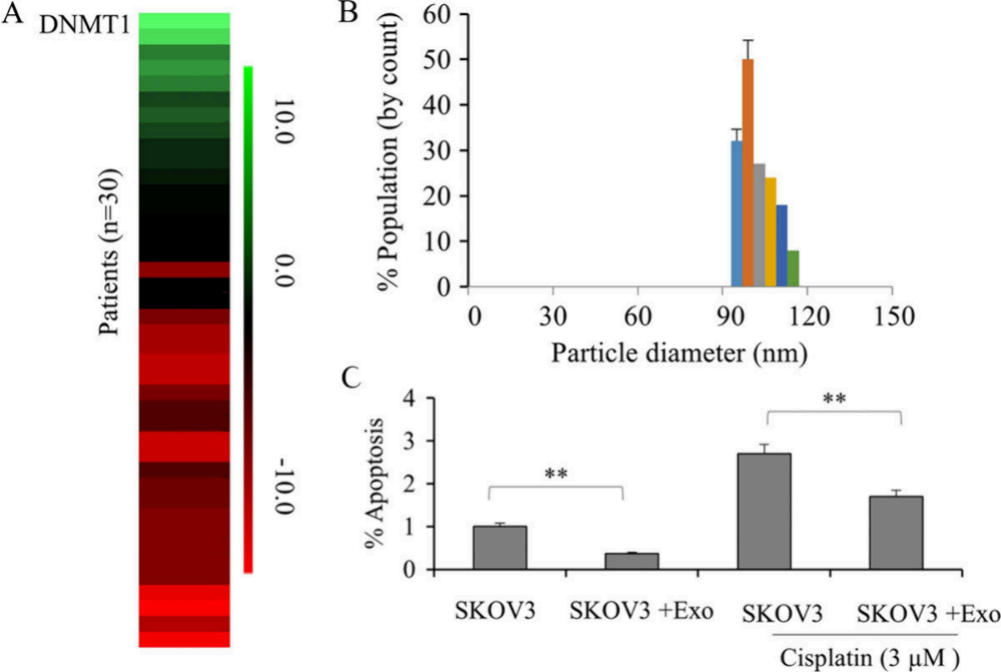
DNMT1 increases ovarian cancer cell cisplatin resistance and lowers apoptosis. (A) The heat map illustrates a significant differential expression of DNMT1 in 30 tissues from ovarian cancer patients compared to surrounding nontumor tissues (*p* < 0.01). (B) The histogram displays the particle diameter (in nanometers) of the tiny vesicles obtained from the ovarian cancer cell line SKOV3 media. (C) DNMT1 overexpression was investigated on cisplatin-induced SKOV3 ovarian cancer cell apoptosis. CCK-8 assay and apoptosis test were used to compare cell survival and apoptosis after treatment with isolated exosomes from SKOV3 cells. Data were stated as mean ± SD from three different experiments (∗∗, *p* < 0.01). Reproduced with permission from ref (61). Copyright 2017 Wiley.

EVs have garnered escalating interest as circulating biomarkers for cancer detection in recent years considering their content and biologic functions. Their potential utility in cancer diagnostics underscores the growing recognition of EVs as promising candidates for enhancing early detection and monitoring of cancer progression. EVs exert a profound influence on tumor development and metastasis by facilitating communication between tumor cells and the intricate networks comprising the tumor microenvironment (TME).^53^ In the context of cancer, EVs intricately regulate various aspects, including immune system modulation and inflammation, cell migration and proliferation, extracellular matrix remodeling, intravasation and extravasation, and tumor cell formation and growth, as well as cancer spreading.^54^ Previous studies have underscored the regulatory role of EVs in viral pathogenesis and the modulation of T cell responses in cancer progression. Notably, antigen-presenting vesicles derived from human and murine B lymphocytes infected with the Epstein–Barr virus have been shown to influence antigen-specific major histocompatibility complex (MHC) class II pathways, thereby impacting cancer development.^55,56^ The presence of EV-associated programmed death ligand 1 (PD-L1) has been implicated in the reduction and suppression of CD8 T cell levels in melanomas, thereby fostering tumor growth and progression.^57^ Integrins, which are intricately linked with EVs, modulate cell behavior to promote metastasis by influencing communication, adhesion, and extracellular matrix (ECM) regulation.^58^ Furthermore, EVs have been found to activate oncogenic pathways, contributing to drug resistance and promoting cancer cell survival, thereby impeding cancer treatment efforts. For instance, exosomes containing lengthy noncoding small nucleolar RNA host genes (SNHGs) have been shown to alter the efficacy of trastuzumab in HER2-positive breast cancer cell lines resistant to trastuzumab treatment.^59,60^ Similarly, ovarian cancer patients exhibiting exosomal DNMT1 transcripts have demonstrated resistance to cisplatin chemotherapy^61^ (Figure 4). Given their extensive involvement in tumor cell development, progression, metastasis, and acquired resistance, EVs hold significant promise as biomarkers and therapeutic tools in the battle against cancer.

## 3 Fundamentals of Optical Biosensors for Cancer Detection

Optical biosensors elucidate the data streams stemming from unique interactions between light and material substrates, manifesting phenomena spanning reflection, refraction, absorption, transmission, interference, fluorescence, and beyond. Within this discourse, the taxonomy of optical biosensors is comprehensively delineated across four primary categories: plasmonic, fluorescence based, fiber based, and colorimetric, each offering distinct modalities for biosensing and analysis.

### 3.1 Plasmonic Optical Biosensors

**Figure 5:**
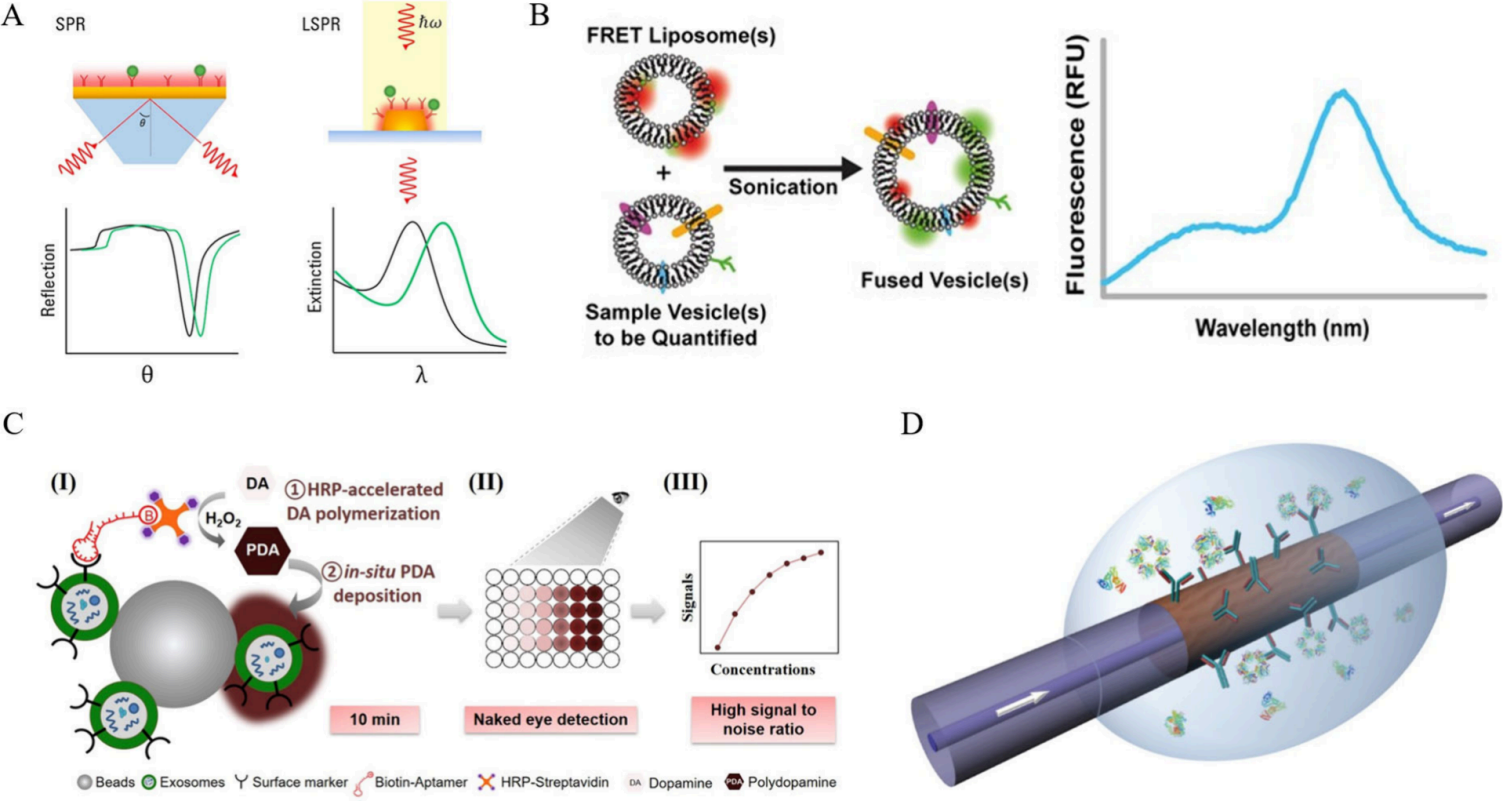
(A) A schematic illustration of SPR and LSPR is demonstrated. Reproduced with permission from ref (154). Copyright 2021 Elsevier. (B) FRET-based quantitative extracellular vesicle detection methodology is presented. Reproduced from ref (82). Copyright 2020 American Chemical Society. (C) Colorimetric detection of exosomes via dopamine to polydopamine conversion is shown. Reproduced with permission from ref (96). Copyright 2020 Elsevier. (D) Optical fiber grating (OFG) technology for biomolecule detection is indicated. From ref (155). CC BY NC-ND 3.0.

Basically, plasmonics examines how light reacts with metals or nanoscaled metal structures, and it is a subfield that combines photonics and electronics. Leveraging optical measurement outcomes, inclusive of alterations in refractive indexes and reflection angles consequent to molecular interactions and binding phenomena upon the plasmonic interface, facilitates probing into pivotal facets pertaining to the concentration, identity, or presence of target molecules.^62^ When polarized light interacts with the plasmonic material, electrons can coherently oscillate at the interface of two different phases, which have different refractive indexes and opposite real parts of the dielectric function.^63−65^ As a result of this phenomenon, surface plasmons (SPs) and surface plasmon polaritons (SPPs) propagating at the surface can be observed. These phenomena play a pivotal part in the advancement of plasmonic sensors, which have eventually led to the development of two main approaches in plasmonic sensing: surface plasmon resonance (SPR) and localized surface plasmon resonance (LSPR) (Figure 5A). At the beginning of the 1900s, the study of SPR technology started with Wood and developed until today with the contributions of Rayleigh, Palmer, and Fano.^66,67^ The first commercial SPR biosensor was produced by Biacore AB in Sweden in 1990 as a uncommon label-free detection method.^68^ Recent studies show that EVs can be detected via SPR to observe cancer cell proliferation and metastasis for a variety of cancer types.^69−71^

In the instance where the detection surface deviates from planarity, instead being adorned with subwavelength noble metallic nanoparticles—typically of gold (Au) or silver (Ag)—facilitating the confinement of SPs proximal to the particles, it denotes the phenomenon of LSPR. Upon illumination of the metallic nanoparticles, a coherent oscillation of electrons ensues, thereby engendering a substantial augmentation in electric field intensity, light absorption, and scattering across the nanostructured milieu. This enhancement is amenable to precise modulation contingent upon the dimensions, morphology, and constituent composition of the nanomaterials employed.^72,73^ The LSPR biosensor monitors changes in the plasmon frequency that are affected by the analyte’s local refractive index. Since the measurements are taken from the close proximity of the nanoparticles, LSPR systems can be designed smaller than planar SPR systems, outshining them in terms of mobility and compactness. This technology has been highly used in biological and chemical detection in the last decades, and promising studies have also been conducted to monitor EV detection for cancer diagnosis.

Another plasmonic-based and nanoparticle-assisted optical sensor method is surface-enhanced Raman spectroscopy (SERS). Raman scattering relies on the energy gain (anti-Stokes) or loss (Stokes) of inelastically dispersed photons caused by vibrational events in molecules. This method presents avenues for in situ and real-time detection, affording insights into molecular structures. However, traditional Raman spectroscopy suffers from limited sensitivity arising from weak Raman scattering and interference signals. By harnessing plasmonic nanostructures, the SERS technique, a subset of Raman scattering, can realize an amplification factor of up to 1 million, thereby markedly enhancing sensitivity and enabling discernment of molecular details with unprecedented precision. Consequently, detection sensitivity can be brought down to the molecular level.^74^ Currently, the SERS method is one of the commonly used optical biosensors for both biomolecules^75^ and EV detection^76,77^ for cancer biomarker identification.

### 3.2 Fluorescence- and Luminescence-Based Optical Biosensors

Luminescence optical biosensors exploit photodiodes or photomultipliers to capture emitted light from luminescent or fluorescent probes, thereby facilitating the elucidation of spatial distribution and binding kinetics of the analyte under investigation. Notably, in recent decades, fluorescence-based instrumentation has undergone exceptional advancements, enabling the sensitive and selective detection of microorganisms, as well as clinical and environmental substances.^78^ The sensitivity of a contemporary fluorometer is as low as a single photon level, and owing to the most cutting-edge super-resolution technology, a fluorescence microscope is able to differentiate between two particles that are separated by less than 10 nm.^79^ Förster resonance energy transfer (FRET) stands out as a prevalent fluorescence sensing technique adept at mitigating the issue of distorted emission signals emanating from biological samples within intricate environments (Figure 5B). Within a FRET system, a donor and an acceptor fluorophore are pivotal components. Specifically, the emission band of the donor fluorophore coincides with the absorption band of the acceptor fluorophore when they are in close spatial proximity.^80^ There are several studies reported in the literature to detect EVs and EV-related biomarkers.^81,82^ Another method is fluorescence correlation spectroscopy (FCS), which relies on tracking the fluctuations in fluorescent signals produced by the labeled molecules diffusing over a confocal detection volume. These alterations may be used to determine the molecular brightness, diffusion coefficient, and quantity of biomolecules that have been fluorescently labeled.^83^ Furthermore, FCS measurements may be used to study the dynamics of biomolecular interactions which could be used to study the binding of specific antibodies to extracellular vesicles.^84^ This enables the possibility of determining the number of certain exosome populations that are present within a heterogeneous mixture via the use of FCS.

### 3.3 Fiber-Based Optical Biosensors

Optical fibers employ a principle known as total internal reflection (TIR) to correlate the detected intensity of light with the concentration of the original target. Target substances can be identified by utilizing immobilized biorecognition molecules, e.g., enzymes, oligonucleotides, or antibodies, attached to the core surface of the fiber. Interactions between analyte and bioreceptor alter the sensitive layer’s characteristics in relation to the analyte concentration which can be identified compared to the initial reference curve.^85^ In recent literature, for instance, fiberoptic-based sensor systems incorporating surface plasmon resonance (SPR), surface-enhanced Raman scattering (SERS), and fluorescence have been introduced. These systems offer several advantages over conventional methods, including reduced equipment costs, portability, remote sensing capabilities, and compact structures. For instance, Zeni et al. developed a portable polymer fiber integrated SPR (FO-SPR) system.^86^ On a parallel track, Yildizhan et al. devised an FO-SPR probe tailored for the detection of extracellular vesicles associated with breast cancer.^87^ Additionally, Li et al. engineered a sandwich-based fluorescence fiber probe enabling rapid, real-time, and quantitative analysis of exosomes.^88^ A particular breed of optical fiber garnering increasing attention is the photonic crystal fiber (PCF), distinguished by a plethora of distinctive attributes potentially advantageous in biosensing applications. The fabrication of photonic crystal fiber involves the uniform distribution of air holes throughout its entire length. There are two main methodologies to guide the light across the fiber: index guiding including a solid core to use TIR optimally and the photonic band gap effect (PBE) incorporating a hollow core fiber.^89^ Modifying the location, dimensions, and arrangement of the cladding holes on the fiber provides a means to optimize sensor efficacy. Xia et al. employed a hollow-core microstructured fiber probe, leveraging Raman scattering sensing for the detection of single exosomes, resulting in markedly heightened sensitivity.^90^ Furthermore, nanostructures or nanostructured coatings can be meticulously crafted onto the fiber surface utilizing the optical fiber grating (OFG) technique (Figure 5D), thereby amplifying the excitation levels of both surface plasmon resonance (SPR) and localized surface plasmon resonance (LSPR) through intensified molecular interactions between the sensing apparatus and analyte constituents.^91^ Chen et al. introduced a fiber Bragg grating sensor tailored for label-free detection of biological molecules.^92^ Another research cohort utilized long-period grating (LPG) probes for the identification of thyroglobulin (TG) protein, a recognized marker indicative of thyroid cancer.^93^

### 3.4 Colorimetric Optical Biosensors

Colorimetric biosensors, which utilize chemoresponsive dyes, provide a color change once they interact with the analyte molecule. Their demonstrated capabilities render them promising candidates for on-site testing platforms, offering visually convenient, straightforward, and user-friendly operation. The sensing mechanisms of colorimetric biosensors utilize the aggregation of nanoparticles^94^ and the catalytic enzyme derived color change.^95^ Colorimetric probes can be employed in device-assisted systems such as paper-based analytical devices (PADs) and lateral flow assays (LFAs), each of which has its own unique advantages. As point-of-care (POC) sensors, they are frequently used due to the several benefits they provide, including their easy fabrication, low cost, technological simplicity, and rapid analysis. To detect cancer-derived exosomes, Xu et al. presented an aptasensor modified with HRP–streptavidin that converts dopamine to brown-colored polydopamine in the presence of hydrogen peroxide (Figure 5C).^96^ Zhang and co-workers used a colorimetric sensor with a plasmonic surface to enhance the sensing features of an exosome detection device.^97^

Selecting the most appropriate optical method for cancer detection necessitates a comprehensive analysis of the specific needs and context of the detection task, including the desired sensitivity, specificity, cost, and practical considerations. Each optical method has its unique strengths and limitations, which are summarized and presented in Table 2, and their suitability can vary depending on the application. Plasmonic biosensors are particularly well-suited for applications requiring high sensitivity and real-time monitoring, such as early detection of cancer biomarkers and studying the interactions within the tumor microenvironment.^71,98^ The high cost and complexity may restrict their use in specialized laboratories and clinical settings. On the other hand, fluorescence-based biosensors are highly effective for applications requiring high sensitivity and detailed imaging, to detect and analyze cancer biomarkers.^99^ They are particularly useful in research settings but may be limited by the complexity and potential for sample damage. Fiber-based biosensors are advantageous for applications requiring portability and real-time monitoring, such as point-of-care testing (POCT) and remote sensing. They offer a balance between sensitivity and practicality but may face challenges related to fragility and alignment. Lastly, colorimetric biosensors are ideal for initial screening and point-of-care testing in resource-limited settings due to their simplicity and low cost.^100^ They are useful for rapid preliminary assessments but may require confirmation with more sensitive methods for accurate diagnosis.

**Table 2:** Advantages and Disadvantages of Optic Biosensors

optical method	advantages	disadvantages
plasmonic optical biosensors	high sensitivity: SPR and LSPR enhance the sensitivity of detection mechanisms, allowing for identification of low concentrations of analytes	high equipment cost: the sophisticated instrumentation required for plasmonic sensing, such as SPR systems, entails significant financial investment
	label-free detection: capable of detecting molecular interactions without additional labeling, preserving the native state of analytes	complex setup and calibration: requires meticulous alignment and calibration to ensure accurate measurements, which can be time-consuming and technically demanding
	real-time monitoring: facilitates continuous observation of molecular binding events, providing instantaneous data on interaction dynamics	environmental sensitivity: highly sensitive to variations in environmental conditions, such as temperature and refractive index changes, which can affect measurement accuracy
	versatility: applicable to a wide range of biomolecular interactions, including those involving proteins, nucleic acids, and lipids	nanoparticle control: the efficacy of LSPR is contingent upon precise control over the size, shape, and distribution of nanoparticles, necessitating advanced fabrication techniques
fluorescence- and luminescence-based optical biosensors	high sensitivity and selectivity: capable of detecting single molecules and providing high specificity in complex biological environments	photobleaching and phototoxicity: fluorescent dyes can degrade under prolonged illumination, reducing signal intensity and potentially damaging biological samples
	super-resolution imaging: advances in fluorescence microscopy enable imaging at resolutions below the diffraction limit, allowing for detailed visualization of subcellular structures	labeling requirement: necessitates the use of fluorescent labels, which may alter the native behavior of the target molecules
	real-time monitoring: techniques such as FRET and FCS allow for the real-time study of dynamic processes and molecular interactions	background fluorescence: endogenous fluorescence from biological samples can interfere with the detection signal, complicating data interpretation
	quantitative analysis: enables precise quantification of analyte concentrations and binding kinetics	complex instrumentation: requires sophisticated and often expensive optical equipment for excitation and detection of fluorescence signals
fiber-based optical biosensors	high sensitivity: utilizes TIR and SPR to achieve high sensitivity in detecting target molecules	fragility: optical fibers are delicate and can be easily damaged during handling or operation
	portability and remote sensing: optical fibers enable the development of compact, portable devices suitable for field applications and remote sensing	alignment and coupling issues: proper alignment and coupling of optical fibers are critical for accurate measurements and can be technically challenging
	cost-effective: offers a lower cost alternative to traditional bulky instrumentation while maintaining high sensitivity	environmental sensitivity: performance can be affected by bending, temperature changes, and other environmental factors
	real-time and in situ monitoring: capable of providing real-time data in various environments, enhancing the applicability in diverse settings	limited multiplexing: generally limited in the ability to simultaneously detect multiple analytes compared to other optical methods
colorimetric optical biosensors	simplicity and user-friendliness: easy to use with a straightforward visual readout, making them accessible for nonspecialist users	limited sensitivity: generally less sensitive compared to other optical methods, which can limit the detection of low-abundance analytes
	cost-effective: inexpensive to fabricate and suitable for large-scale production, ideal for POCT and resource-limited settings	subjective interpretation: visual color changes can be subjective and may lead to inconsistent results among different users
	rapid analysis: provides quick results without the need for complex instrumentation or extensive sample preparation	false positives/negatives: susceptible to interferences that can cause erroneous results, reducing reliability
	versatile applications: adaptable to various formats, such as paper-based analytical devices (PADs) and lateral flow assays (LFAs)	limited dynamic range: may not be suitable for detecting a wide range of analyte concentrations, requiring complementary methods for comprehensive analysis

## 4 Applications in Cancer Diagnosis through Extracellular Cellular Detection

Early cancer screening improves intervention and reduces mortality in healthy and high-risk individuals. Imaging and tissue biopsy usually confirm solid tumor diagnoses. Biopsies may not correctly represent tumor heterogeneity since they depend on the sample location.^101^ Tissue biopsy for prognostic monitoring is too intrusive. Traditional tumor indicators are not clinically useful. A unique noninvasive detection method that can fully characterize tumors, enable early cancer screening, and properly evaluate treatment efficacy is needed.^102^ It is widely acknowledged that the growth of cancerous tumors results in the release of diverse constituents into the bloodstream.^103^ These constituents encompass circulating tumor cells (CTCs), tumor-derived DNA (ctDNA), various EVs, proteins, and metabolites.^104^ In recent studies, there has been a notable emphasis on investigating EVs to develop varieties of diagnostic techniques further. One notable characteristic of EVs is their presence in diverse bodily fluids.^105^

In contrast to conventional tissue biopsies, which necessitate intricate sampling methods and are invasive, liquid biopsies utilizing EVs provide several advantages. These include the ability to monitor tumor progression over time, assess long-term therapy response, facilitate recurrent sampling, and enable simplified sampling management. In addition, it is worth noting that conventional biopsies that rely on symptom presentation may be inadequate in identifying early stage cancer because of the delayed manifestation of some symptoms. Conversely, EV-based liquid biopsy, operating at the genetic level, has the potential to overcome the constraints associated with symptom-based approaches and enable the early detection of cancer.^106^

The established techniques employed for the identification and characterization of EVs consist of flow cytometry^107,108^ enzyme-linked immunosorbent assay (ELISA),^108,109^ tunable resistive pulse sensing (TRPS)^110,111^ dynamic light scattering (DLS),^112,113^ nanoparticle tracking analysis (NTA),^50,114^ Western blot,^115,116^ and real-time polymerase chain reaction (PCR).^117−119^ Furthermore, recent advancements in high-sensitivity detection techniques have facilitated the attainment of specific procedures to achieve a level of a single EV.^120^ Biosensors have been employed in medical and biological areas worldwide for decades as fast, dependable, and precise analytical procedures. Recently, biosensors and nanobiosensors have been investigated for EV detection and quantification.^6^ Optical^111,121^ and electrochemical^122−125^ methods are used to build EV biosensors. Most research has employed nanomaterials to improve biosensor accuracy and sensitivity to detect low EV concentrations. In this discipline, discovering detection methods for high-throughput screening, low limit of detection (LOD), real-time analysis, and minimal sample volume is crucial. Currently, optical techniques have demonstrated exceptional precision and reliability in the measurement of biological entities.^126^ Various optical methods, including fluorescence,^127,128^ Raman scattering,^129,130^ surface plasmon resonance (SPR),^131,132^ and colorimetry,^133,134^ have been utilized for the quantification of exosomal proteins or miRNAs.^126^ This section aims to provide a comprehensive overview of the existing literature on cancer diagnosis using EVs.

Plasmonic biosensor applications for EV detection to cancer diagnosis constitute the majority of the literature in terms of fast, label-free, and real-time detection. Some certain examples of plasmonic EV detection for cancer diagnosis are mentioned below, and a comprehensive table is given in Table 3.

**Table 3:** Plasmonic Biosensor Applications to Detect EVs for Cancer Diagnostics

extracellular vesicle source	specific target	isolation method	detection method	source
ovarian cancer (OV90, OVCAR3, CaOV3), ovarian benign (TiOSE4) cell lines	CD63, EpCAM, CD24	SEC	electrokinetically enhanced yield of plasmonic sensing (KeyPLEX)	(166)
blood sample	N/A	SEC	SERS	(167)
healthy epithelial (A549) and breast cancer (MCF-7) cell lines	CD63	ultrafiltration	concentric gradient nanoplasmonic sensor	(168)
breast cancer (MCF-7) and normal breast (MCF-10A) cell lines	aptamer (T_30_ and A_30_)	ultracentrifuge	SPR	(70)
glioblastoma cell line (U87-MG)	CD9 and CD81	ultracentrifuge	SPR	(169)
human NSCLC (A549), human normal bronchial epithelial (BEAS-2B), breast cancer (MDA-MB-231), and normal breast epithelial (MCF-10A) cells	EGFR and LG3BP	ultracentrifuge	SPR	(170)
breast cancer cell lines (MCF-7, SKBR-3, MDA-MB-231, BT474)	MUC1, HER2, and CEA	ultracentrifuge	SERS	(149)
breast cancer cell lines (MCF-7, SKBR-3, MDA-MB-231, BT474)	N/A	SEC	SERS	(150)
fibroblast L cells	EpCAM	centrifugation	LSPR	(171)
nonsmall cell lung cancer (A549, H460, and H1299) and normal lung (BEAS-2B) cell lines	CD63, CD81, EpCAM, EGFR	dUC	SPRi	(172)

The present liquid biopsy method is unable to achieve marker-free diagnosis due to the absence of clinically validated markers specific for cancer stem cells (CSC). Additionally, relying on EV separation in existing technologies undermines the clinical significance of EV-based liquid biopsy.^135^ In a study, CSC-EVs were used as a separate liquid biopsy approach, which created new 3D sensors containing self-functionalized nanoscale probes.^136^ These sensors have the capability to perform surface-enhanced Raman scattering (SERS), allowing for comprehensive molecular and functional characterization of challenging-to-identify CSC EVs. Significant enhancements were seen in the SERS signals, resulting in a notable reduction in the LOD of 1 EV/1 μL. A preliminary evaluation of the efficacy of this procedure was subsequently carried out to validate its use in cancer diagnosis and identification of the specific tissue from which the disease originated. The performance of an artificial neural network in discriminating between cancerous and noncancerous cases exhibited perfect sensitivity and specificity, reaching 100% for three challenging types of cancer (breast, lung, and colorectal). The binary classification task successfully obtained a 100% accuracy rate in differentiating one specific origin tissue from others. On the other hand, the multiclass classification task, which aimed to identify three different origin tissues simultaneously, achieved an accuracy rate of up to 79%. This noninvasive instrument possesses the capacity to enhance current methods of cancer diagnosis, monitoring of treatment progress, and long-term disease monitoring by means of validation using a substantial cohort of clinical samples.

For SERS characterization of ovarian and endometrial cancer EVs, another study presented a plasmonic scaffold made of a microscale biosilicate substrate loaded with silver nanoparticles.^137^ Fast and inexpensive production of these substrates is possible without the need for sophisticated machinery or lithography. To begin the process of making substrates, cysteamine was applied to metal surfaces in order to nonspecifically attract EVs to SERS hotspots. To avoid the localization of complementary chemical characteristics (lipids/proteins), effective chemical treatments were performed around the metal-enriched portions of the EVs’ surfaces to facilitate the enzymatic cleavage of extraluminal moieties. After the extraluminal domain of EVs is enzymatically cleaved, their susceptibility to ovarian and endometrial cancers is significantly reduced. An appropriate technique for fast and label-free evaluation and quantification of the great heterogeneity of EVs extracted from clinical samples has been established with this platform.

In a different study, the development of a compact SPR biosensor capable of detecting exosomal EGFR and PD-L1 biomarkers with high sensitivity was highlighted. This system was reported to reach a resolution of 8.311 × 10^–6^ RIU and a sensitivity of 9.258 × 10^3^%/RIU, which is more sensitive than ELISA.^71^ This system presented a cost-efficient and user-friendly structure that is more suitable for clinical applications. Thakur et al. presented a self-assembled monolayer gold nanoisland modified LSPR (SAM-AuNls LSPR) platform for the detection of MVs derived from A-549 lung cancer cells, SH-SY5Y neuroblastoma cells, blood serum, and urine from a lung cancer mouse model.^138^ It was reported that the sensor can reach 0.194 μg/mL sensitivity and differentiate cancer EV types. Liang and co-workers made a label-free plasmonic metasurface to detect prostate specific antigen (PSA) for the early detection of prostate cancer (PCa).^139^ This portable immunoassay system was reported to complete detection in 20 min and differentiate early stages and benign stages of the PCa. Recently, a portable SPR sensor study was conducted that was modified with three different layers consisting of Au mirror/SiO_2_ spacer/Au nanohole to enhance sensitivity.^140^ EpCAM and CD63 aptamers were immobilized on the sensor surface to separate HepG2 hepatocellular carcinoma cells and L02 healthy-cell-derived EVs.

**Figure 6:**
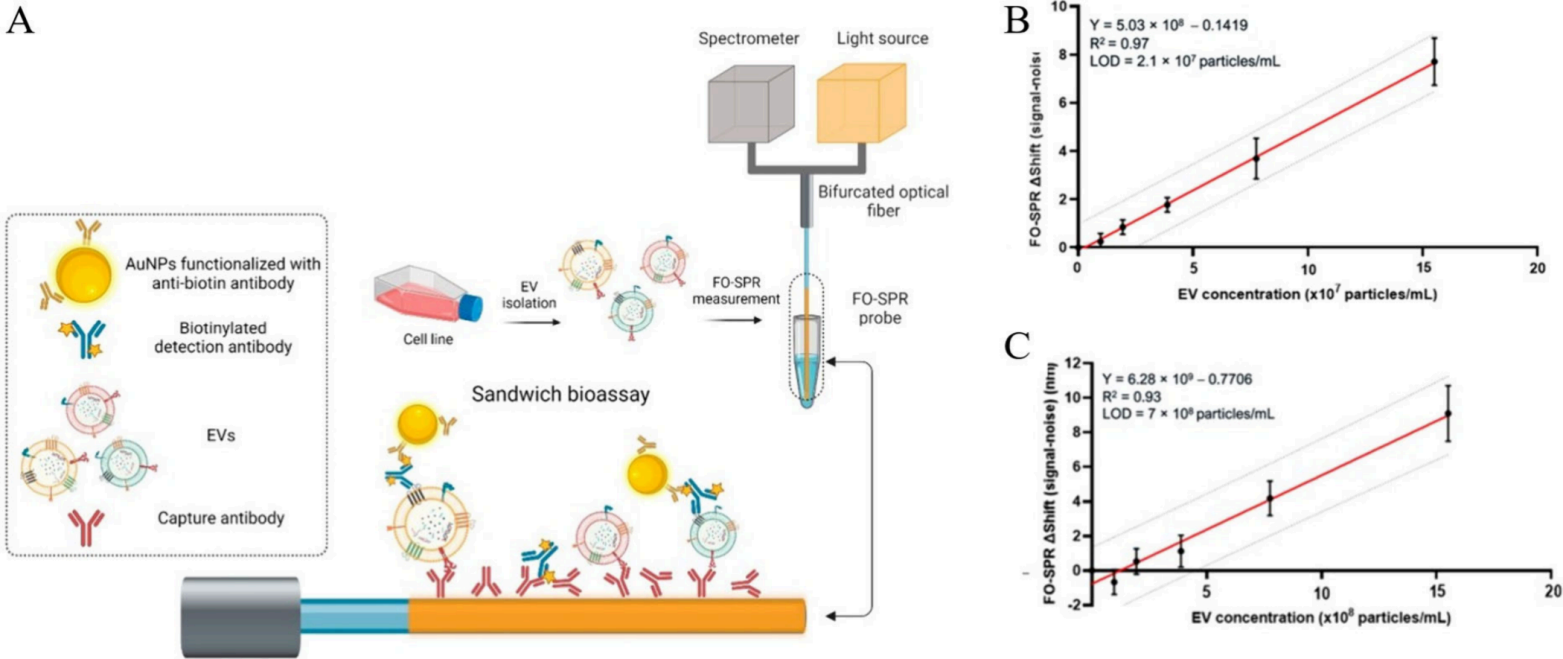
(A) Schematic of the FO-SPR EV detection sandwich bioassay, illustrating specific capture of EVs (from SK-BR-3 or MCF7 cell lines) by immobilized capture antibodies (anti-HER2 or anti-EpCAM). Biotinylated detection antibodies (^B^anti-CD9, ^B^anti-CD63, or ^B^anti-CD81) identify CD9, CD63, and CD81 tetraspanins on EV surfaces, followed by signal amplification using AuNPs functionalized with antibiotin antibodies. Antibodies on FO-SPR and AuNPs have random orientations, and EVs are represented in varied sizes and colors, reflecting biological heterogeneity. (B) FO-SPR detection of SK-BR-3 EV concentrations in a detection buffer (50 mM MES pH 6, 0.01% BSA, 0.01% Tween 20) using anti-HER2/^B^anti-CD9 antibodies. (C) FO-SPR detection of SK-BR-3 EV concentrations in 100-fold diluted pooled plasma using anti-HER2/^B^anti-CD9 antibodies. Signal values derived by subtracting the negative control (0 particles/mL). From ref (87). CC BY 4.0.

The integration of fiber optic technology with plasmonic biosensors is a cutting-edge approach designed to enhance sensor sensitivity. A fiberoptic-integrated surface plasmon resonance (FO-SPR) biosensor was created for the purpose of detecting breast cancer-specific EVs directly from blood plasma.^87^ To detect SK-BR-3 EVs, FO-SPR probes were functionalized with anti-HER2/biotinylated anti-CD9 (^B^anti CD9) antibodies to produce a sandwich bioassay (Figure 6A). The detection of EVs was possible with the use of this antibody combination, with the LOD values being 2.1 × 10^7^ particles/mL in buffer solution and 7 × 10^8^ particles/mL in blood plasma (Figure 6B,C). Following that, the bioassay underwent modifications in order to identify MCF7 EVs in blood plasma. This was accomplished by utilizing an anti-EpCAM/^B^antimix combination, which resulted in an LOD of 1.1 × 10^8^ particles/mL. Furthermore, as for the bioassay’s specificity, it was able to detect no signal in plasma samples taken from 10 healthy people who had not yet been diagnosed with breast cancer. In another study, WS_2_-supported gold nanobipyramid (Au NBPs) modified optical microfibers were utilized for highly sensitive LSPR applications to detect prostate cancer exosomes.^141^ These microfibers enhanced the plasmonic properties, and the LOD of the sensor improved up to 23.5 particles mL^–1^ in phosphate buffered saline (PBS) solution, which is 2 orders of magnitude more sensitive than previously reported data. This was reported as 570.6 particles mL^–1^ in 10% serum.

Moreover, a refractive index (RI) based sensor that is extremely sensitive has been created, expanding on the success of liquid biopsy in detecting cancer with EVs in bodily fluid.^142^ This sensor was built on a small, high-index coated polymer waveguide Bragg grating that includes metal below the covering. A notable improvement in RI sensitivity and dynamic range was seen due to the synergistic impact of the high-index coating with the metal under the coating. Based on the results of the combined finite element method and coupled-mode-theory analyses, the proposed sensor has a sensitivity of 408–861 nm/RIU across a wide dynamic range of 1.32–1.44 and a strong evanescent field within 150 nm of the waveguide surface that is in accordance with the EV size. The suggested device is an appealing choice for early stage cancer detection in real time and on-chip.

**Figure 7:**
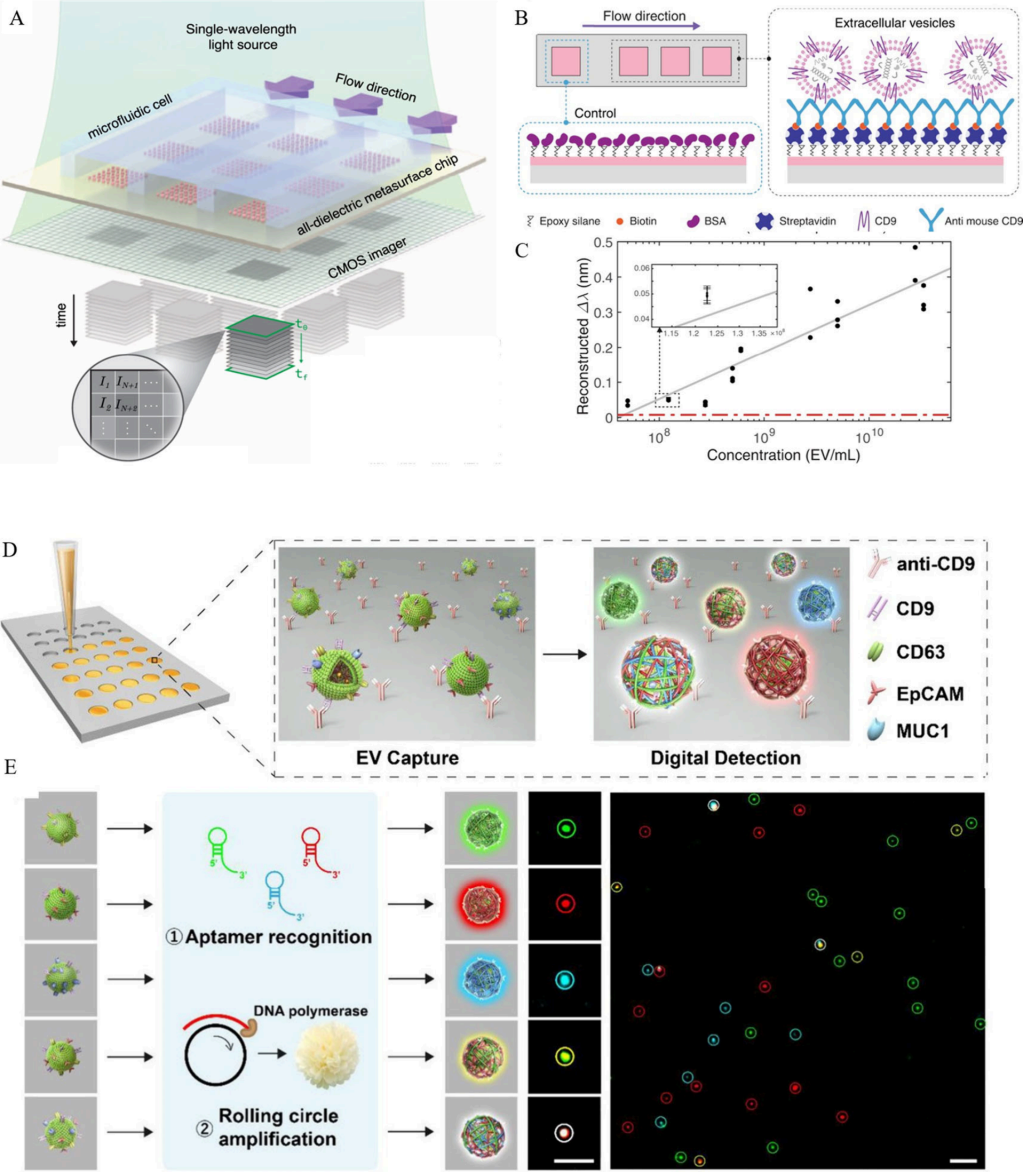
(A) A schematic representation of a real-time in-flow imaging platform is depicted with a 2D microarray with all-dielectric sensors coupled with a microfluidic cell with three flow channels. The metasurface chip is illuminated using a single-wavelength light source and captured by a large-area CMOS camera, enabling the acquisition of high-resolution intensity maps (*I*_1_, *I*_2_, ..., *I*_*N*+1_, *I*_*N*+_2, ...) corresponding to individual sensors (*I* refers to intensity of the sensors in pixel resolution). (B) An illustration of a biorecognition assay designed for the detection of EVs, explaining the interaction with detection sensors and a control sensor preblocked with bovine serum albumin (BSA) to mitigate nonspecific binding. (C) The reconstructed spectral shift calibration curve of EVs is presented (inset: a magnified *x*-axis to enhance the resolution of small error bars for a representative data point). From ref (121). CC BY 4.0. (D) An overview of the digital plasmonic photothermal imaging and enumeration assay steps is presented. The process involves the capture of plasma extracellular EVs on the surface of an anti-CD9-engineered biochip. The distinct signals observed are attributed to the variety of surface proteins exhibited by the captured EVs. (E) The captured EVs are labeled with DNA aptamers, followed by rolling circle amplification to produce localized amplified fluorescent signals. The resulting signals are then visualized using confocal microscopy, with fluorescence images (CD63, green; EpCAM, red; and MUC1, blue) Scale bar: 3 μm. Reprinted with permission from ref (143). Copyright 2020 Wiley.

A recent study focused on the development of nanophotonic biosensors that employ single-wavelength imaging techniques to attain heightened sensitivity.^121^ The process of reconstructing the spectral shift accomplishes the aforementioned objective, hence preventing the necessity for laborious wavelength scanning and the utilization of spectrometers. This was achieved using quasi-bound states in continuous (BIC) modes, which exhibit high-Q resonances of “diatomic metasurfaces”. This method broadens BIC beyond asymmetric metasurfaces. Changing a meta-atom’s ellipticity purposefully disrupts dimer symmetry. This alteration gives the metamolecule high-Q resonance and allows the electromagnetic field and analyte to overlap. Diatomic structures simplify manufacturing and are better for biosensing because they can design in-plane asymmetry more easily than single-unit metasurfaces. These metasurfaces were combined with advanced data processing methods for imaging biosensing as shown in Figure 7A. Real-time wide-area intensity metasurface images are captured by the biosensor using a single wavelength. These images are analyzed using linear estimation to precisely reproduce spectral shift data. The integration of antibody-functionalized metasurface chips with microfluidics (Figure 7B) in a 2D microarray configuration enabled the identification of breast cancer EVs in a flowing system, allowing for real-time monitoring of their binding. These vesicles are significant biomarkers for diagnostic purposes, as they are closely associated with the pathology of the illness. The optofluidic sensor facilitated the identification of an average of 0.41 nanoparticle/μm^2^ and the immediate measurement of attached extracellular vesicles from solutions with concentrations as low as 1.23 × 10^8^ particles/mL as depicted in Figure 7C.

Fluorescent and colorimetric sensors are also commonly used sensor types for EV detection, considering cost-effective, rapid, and straightforward analysis. Table 4 presents recent studies regarding cancer diagnosis. Within the scope of another research, a localized fluorescent imaging technique was created to analyze several proteins within each EV.^143^ EVs were extracted from the clinical plasma sample using a biochip engineered with anti-CD9 antibody. Subsequently, the EVs collected underwent particular identification by utilizing various DNA aptamers (CD63/EpCAM/MUC1) as shown in Figure 7D. This was followed by the implementation of rolling circle amplification, which produced localized fluorescent signals (Figure 7E). Through the examination of the diversity inside individual EVs, the use of high-dimensional data obtained from each EV holds the potential to offer more accurate insights compared to bulk quantification methods such as ELISA. Specifically, the evaluation of the proportion of CD63/EpCAM/MUC1-triple positive EVs in breast cancer can be enhanced through the analysis of these EVs at an individual level. Additional information about EVs may be gathered using this particular EV heterogeneity analysis technique to accomplish multiple cancer diagnoses and categorizations.

**Table 4:** Colorimetric and Fluorescence Biosensor Applications to Detect EVs for Cancer Diagnostics

extracellular vesicle source	specific target	isolation method	detection method	ref
human placental cell line (BeWo)	PLAP, CD63	gold-loaded nanoporous ferric oxide nanozymes	colorimetric	(47)
human breast cancer cell line (MDA-MB-231)	PD-L1	ultracentrifuge	fluorescent aptasensor	(173)
MDA-MB-231, MCF-10A, and MCF-7	CD63	ultracentrifuge	colorimetric and fluorescent	(133)
human colorectal adenocarcinoma (HT-29) and diploid human (WI-38) cells	EpCAM	immunoaffinity/flow cytometry	colorimetric aptasensor	(174)
HeLa cells and serum samples	CD63	immunoaffinity	fluorescence	(175)
nasopharyngeal carcinoma (NPC) cells and plasma samples from NPC patients	LMP1, EGFR	magnetic separation	AuNP-based colorimetric assay	(176)
breast cancer cells (MCF-7)	CD63	ultracentrifugation	colorimetric	(177)

Globally, colorectal cancer (CRC) ranks fourth in incidence and third in cancer-related deaths. The gold standard for colorectal cancer detection, colonoscopy, is also the most invasive and costly treatment when it comes to population screenings.^144^ Although it has poor specificity, fecal occult blood tests are now utilized extensively for CRC screening. In particular, compared to normal colon fibroblast cell lines, CRC cell lines express a far larger quantity of CD147 on circulating EVs. Based on these findings, a noninvasive method for detecting CRC by CD147-positive EVs was devised: a quantitative lateral flow immunoassay test that uses magnetic nanoparticles as labels connected to an inductive sensor.^145^ Indications from the findings of the CD147 antigen quantification in EVs extracted from plasma suggest that this device, with its user-friendly design and quick reaction time, can be utilized as a point-of-care tool for colorectal cancer screening or treatment monitoring.

In order to enhance the comprehension of the mechanistic dynamics between EVs and cells, it is imperative to employ additional techniques that can generate label-free and real-time kinetic data pertaining to EV uptake and EV properties.^146^ In essence, the use of single-vesicle approaches will serve as the fundamental basis for elucidating a cellular communication paradigm that is constructed upon an EV-based network.^147^ Currently, a significant obstacle faced by EVs is the need to effectively manage the heterogeneity among EV populations.^148^ To promote this goal, optical methods such as nanophotonics based, refractive index based, SERS, fluorescence, and SPR have been used recently. The working principles, advantages, and disadvantages of these methods are discussed in detail in the previous sections. Here, we discuss the aforementioned optical approaches extensively, providing examples to show how they might be used for EV detection. Recent studies support that artificial intelligence (AI) assisted strategies were also frequently combined with biosensor applications. Different studies utilized diverse AI strategies, such as artificial neural networks (ANNs), to diagnose cancer and decide cancer stages.^149,150^ While optical detection methods for EVs offer significant promise, there are technical and biological challenges that must be addressed to improve their clinical applicability. Optical methods must achieve higher sensitivity and specificity to detect low concentrations of EVs and accurately differentiate between cancerous and noncancerous cells from complex biological samples. Literature shows that the LOD may decrease when the patient sample is used.^141^ Effective EV isolation is also critical for accurate detection and analysis. Current methods like ultracentrifugation and size-exclusion chromatography can be time-consuming and may not efficiently separate EVs from other components in the sample as mentioned in the previous sections. For biological human samples, preprocessing may be needed before EV isolation and this should not damage target biomarkers. Moreover, most of the isolation techniques require certain setups and cannot be replaced and moved, which might be problematic for clinical applications.^151^ In summary, while optical detection methods for EVs in cancer diagnosis show great potential, addressing the technical and biological challenges is crucial for their successful clinical application. Continued research and development in this field, along with the integration of AI and other advanced technologies, will likely lead to significant improvements in early cancer detection and patient outcomes.

## 5 Conclusion

Optical biosensors play a pivotal role in advancing the utilization of EVs as clinical diagnostic tools for cancer, notably by enhancing detection sensitivity down to the single EV level and enabling high-throughput screening of cancer-specific molecular signatures harbored within EVs. This review delineates the classification and isolation techniques employed for EVs, elucidates the various types of optical biosensors utilized for quantifying and identifying cancer-specific EVs, and delves into the prospective clinical applications of optical biosensors through EV analysis at the POC settings. Moreover, the review conducts a comparative analysis between conventional methods for EV characterization and identification and the novel approaches offered by optical biosensors, encompassing both their respective advantages and limitations.

The review denotes an overview of several optical biosensor methodologies, including SPR, FO-SPR, SERS, imaging-based, fluorescence, FRET, FCS, and colorimetric assays. These diverse methods offer critical insights into determining the size, shape, concentration, and molecular composition of the biomolecular cargo within EVs. In comparison to alternative biosensor modalities such as electrochemical and piezoelectric techniques, optical biosensors offer distinct advantages. Notably, they afford high sensitivity, enabling detection at low concentrations, real-time monitoring capabilities, facilitating dynamic analysis, high-throughput processing, allowing for rapid screening of large sample sets, and minimal sample volume consumption, conserving precious biological materials. These attributes collectively underscore the significance of optical biosensors in the realm of EV-based diagnostics and research endeavors.

Indeed, a notable challenge in the realm of optical biosensors lies in the expense associated with the detectors utilized for tracking spectral changes in resonance peaks. However, recent advancements in imaging methodologies have introduced spectrometer-less measurement techniques, leading to a significant reduction in the overall cost of optical platforms. While this development is instrumental in promoting affordability, particularly crucial for POC applications involving EVs, maintaining high sensitivity concurrently poses a formidable obstacle. Balancing the imperative of cost-effectiveness with the necessity for heightened sensitivity presents a multifaceted challenge. Nevertheless, ongoing research endeavors aim to address this conundrum by exploring innovative approaches to enhance sensitivity without compromising affordability. Such efforts underscore the dynamic landscape of optical biosensing technology, driven by the pursuit of solutions that reconcile competing demands for cost efficiency and analytical performance.

The initial challenge in analyzing EVs within liquid biopsies involves the intricate heterogeneity of EV subpopulations. This complexity necessitates the integration of novel isolation techniques, such as microfluidic platforms, to classify EV subtypes accurately. The isolation of distinct EV subtypes holds promise for refining our understanding of cancer pathology and physiology. Moreover, integrating isolation methods with biosensors within a single platform offers the potential for rapid and cost-effective EV analysis. Another obstacle in EV analysis pertains to determining the tissue of origin for tumors. While isolated EV subpopulations provide insights into disease modeling, tracking single EVs offers a more comprehensive understanding of clinical sample heterogeneity. Consequently, researchers are implementing sophisticated techniques, such as multilayered noble-metal nanoparticles in SERS templates or fluorescence methods, for the digital detection of individual EVs. By leveraging high-throughput analysis methods and advanced data analysis techniques like machine learning and deep learning, the molecular fingerprints of individual EVs can be elucidated, enabling the identification of tumor-originating tissue.^152^ Such advancements hold significant promise for the advancement of personalized medicine and the development of therapeutic agents. In summary, optical biosensors are expected to cultivate the clinical EV research by decreasing fabrication and instrument related costs, integrating novel isolation methods, providing single EV sensitivity, and applying comprehensive data analysis methods for disease modeling.

## References

[ref1] Kadakia K. C.; Hui D.; Chisholm G. B.; Frisbee-Hume S. E.; Williams J. L.; Bruera E. Cancer Patients’ Perceptions Regarding the Value of the Physical Examination: A Survey Study. Cancer 2014, 120 (14), 2215–2221. 10.1002/cncr.28680. 24899511 PMC5841459

[ref2] Son M. H.; Park S. W.; Sagong H. Y.; Jung Y. K. Recent Advances in Electrochemical and Optical Biosensors for Cancer Biomarker Detection. BioChip J. 2023, 17 (1), 44–67. 10.1007/s13206-022-00089-6.

[ref3] Galvão-Lima L. J.; Morais A. H. F.; Valentim R. A. M.; Barreto E. J. S. S. MiRNAs as Biomarkers for Early Cancer Detection and Their Application in the Development of New Diagnostic Tools. Biomed. Eng. Online 2021, 20 (1), 21 10.1186/s12938-021-00857-9. 33593374 PMC7885381

[ref4] Wu J.; Hu S.; Zhang L.; Xin J.; Sun C.; Wang L.; Ding K.; Wang B. Tumor Circulome in the Liquid Biopsies for Cancer Diagnosis and Prognosis. Theranostics 2020, 10 (10), 4544–4556. 10.7150/thno.40532. 32292514 PMC7150480

[ref5] Chowdhury T.; Cressiot B.; Parisi C.; Smolyakov G.; Thiébot B.; Trichet L.; Fernandes F. M.; Pelta J.; Manivet P. Circulating Tumor Cells in Cancer Diagnostics and Prognostics by Single-Molecule and Single-Cell Characterization. ACS Sensors 2023, 8 (2), 406–426. 10.1021/acssensors.2c02308. 36696289

[ref6] Martín-Gracia B.; Martín-Barreiro A.; Cuestas-Ayllón C.; Grazú V.; Line A.; Llorente A.; de la Fuente J. M.; Moros M. Nanoparticle-Based Biosensors for Detection of Extracellular Vesicles in Liquid Biopsies. J. Mater. Chem. B 2020, 8 (31), 6710–6738. 10.1039/D0TB00861C. 32627783

[ref7] Rolfo C.; Russo A. Liquid Biopsy for Early Stage Lung Cancer Moves Ever Closer. Nat. Rev. Clin. Oncol. 2020, 17 (9), 523–524. 10.1038/s41571-020-0393-z. 32457540

[ref8] Kholafazad Kordasht H.; Hasanzadeh M. Biomedical Analysis of Exosomes Using Biosensing Methods: Recent Progress. Anal. Methods 2020, 12 (22), 2795–2811. 10.1039/D0AY00722F. 32930202

[ref9] Nguyen C. M.; Sallam M.; Islam M. S.; Clack K.; Soda N.; Nguyen N.-T.; Shiddiky M. J. A. Placental Exosomes as Biomarkers for Maternal Diseases: Current Advances in Isolation, Characterization, and Detection. ACS sensors 2023, 8 (7), 2493–2513. 10.1021/acssensors.3c00689. 37449399

[ref10] Afridi W. A.; Strachan S.; Kasetsirikul S.; Pannu A. S.; Soda N.; Gough D.; Nguyen N.-T.; Shiddiky M. J. A. Potential Avenues for Exosomal Isolation and Detection Methods to Enhance Small-Cell Lung Cancer Analysis. ACS Meas. Sci. Au 2023, 3 (3), 143–161. 10.1021/acsmeasuresciau.2c00068. 37360040 PMC10288614

[ref11] Ma Z.; Xu H.; Ye B.-C. Recent Progress in Quantitative Technologies for the Analysis of Cancer-Related Exosome Proteins. Analyst 2023, 148 (20), 4954–4966. 10.1039/D3AN01228J. 37721099

[ref12] Tokel O.; Inci F.; Demirci U. Advances in Plasmonic Technologies for Point of Care Applications. Chem. Rev. 2014, 114 (11), 5728–5752. 10.1021/cr4000623. 24745365 PMC4086846

[ref13] Tokel O.; Yildiz U. H.; Inci F.; Durmus N. G.; Ekiz O. O.; Turker B.; Cetin C.; Rao S.; Sridhar K.; Natarajan N.; Shafiee H.; Dana A.; Demirci U. Portable Microfluidic Integrated Plasmonic Platform for Pathogen Detection. Sci. Rep. 2015, 5 (1), 9152 10.1038/srep09152. 25801042 PMC4371189

[ref14] Saylan Y.; Erdem Ö.; Inci F.; Denizli A. Advances in Biomimetic Systems for Molecular Recognition and Biosensing. Biomimetics 2020, 5 (2), 20 10.3390/biomimetics5020020. 32408710 PMC7345028

[ref15] Inci F.; Karaaslan M. G.; Mataji-Kojouri A.; Shah P. A.; Saylan Y.; Zeng Y.; Avadhani A.; Sinclair R.; Lau D. T.-Y.; Demirci U. Enhancing the Nanoplasmonic Signal by a Nanoparticle Sandwiching Strategy to Detect Viruses. Appl. Mater. Today 2020, 20, 100709 10.1016/j.apmt.2020.100709.

[ref16] Lu X.; Yao C.; Sun L.; Li Z. Plasmon-Enhanced Biosensors for MicroRNA Analysis and Cancer Diagnosis. Biosens. Bioelectron. 2022, 203, 114041 10.1016/j.bios.2022.114041. 35121447

[ref17] Li H.; Huang T.; Lu L.; Yuan H.; Zhang L.; Wang H.; Yu B. Ultrasensitive Detection of Exosomes Using an Optical Microfiber Decorated with Plasmonic MoSe2-Supported Gold Nanorod Nanointerfaces. ACS Sensors 2022, 7 (7), 1926–1935. 10.1021/acssensors.2c00598. 35761169

[ref18] Yáñez-Mó M.; Siljander P. R. M.; Andreu Z.; Zavec A. B.; Borràs F. E.; Buzas E. I.; Buzas K.; Casal E.; Cappello F.; Carvalho J.; Colás E.; Cordeiro-Da Silva A.; Fais S.; Falcon-Perez J. M.; Ghobrial I. M.; Giebel B.; Gimona M.; Graner M.; Gursel I.; Gursel M.; Heegaard N. H. H.; Hendrix A.; Kierulf P.; Kokubun K.; Kosanovic M.; Kralj-Iglic V.; Krämer-Albers E. M.; Laitinen S.; Lässer C.; Lener T.; Ligeti E.; Line A.; Lipps G.; Llorente A.; Lötvall J.; Manček-Keber M.; Marcilla A.; Mittelbrunn M.; Nazarenko I.; Nolte-’t Hoen E. N. M.; Nyman T. A.; O’Driscoll L.; Olivan M.; Oliveira C.; Pállinger É.; Del Portillo H. A.; Reventós J.; Rigau M.; Rohde E.; Sammar M.; Sánchez-Madrid F.; Santarém N.; Schallmoser K.; Ostenfeld M. S.; Stoorvogel W.; Stukelj R.; Van Der Grein S. G.; Helena Vasconcelos M.; Wauben M. H. M.; De Wever O. Biological Properties of Extracellular Vesicles and Their Physiological Functions. J. Extracell. Vesicles 2015, 4, 27066 10.3402/jev.v4.27066. 25979354 PMC4433489

[ref19] Li B.; Cao Y.; Sun M.; Feng H. Expression, Regulation, and Function of Exosome-Derived MiRNAs in Cancer Progression and Therapy. FASEB J. 2021, 35 (10), e21916 10.1096/fj.202100294RR. 34510546

[ref20] Boriachek K.; Islam M. N.; Möller A.; Salomon C.; Nguyen N.-T.; Hossain M. S. A.; Yamauchi Y.; Shiddiky M. J. A. Biological Functions and Current Advances in Isolation and Detection Strategies for Exosome Nanovesicles. Small 2018, 14 (6), 1702153 10.1002/smll.201702153. 29282861

[ref21] Lai J. J.; Chau Z. L.; Chen S.-Y.; Hill J. J.; Korpany K. V.; Liang N.-W.; Lin L.-H.; Lin Y.-H.; Liu J. K.; Liu Y.-C.; Lunde R.; Shen W.-T. Exosome Processing and Characterization Approaches for Research and Technology Development. Adv. Sci. 2022, 9 (15), 2103222 10.1002/advs.202103222. PMC9130923 35332686

[ref22] Welsh J. A.; Goberdhan D. C. I.; O’Driscoll L.; Buzas E. I.; Blenkiron C.; Bussolati B.; Cai H.; Di Vizio D.; Driedonks T. A. P.; Erdbrügger U.; Falcon-Perez J. M.; Fu Q.-L.; Hill A. F.; Lenassi M.; Lim S. K.; Mahoney M. G.; Mohanty S.; Möller A.; Nieuwland R.; Ochiya T.; Sahoo S.; Torrecilhas A. C.; Zheng L.; Zijlstra A.; Abuelreich S.; Bagabas R.; Bergese P.; Bridges E. M.; Brucale M.; Burger D.; Carney R. P.; Cocucci E.; Crescitelli R.; Hanser E.; Harris A. L.; Haughey N. J.; Hendrix A.; Ivanov A. R.; Jovanovic-Talisman T.; Kruh-Garcia N. A.; Ku’ulei-Lyn Faustino V.; Kyburz D.; Lässer C.; Lennon K. M.; Lötvall J.; Maddox A. L.; Martens-Uzunova E. S.; Mizenko R. R.; Newman L. A.; Ridolfi A.; Rohde E.; Rojalin T.; Rowland A.; Saftics A.; Sandau U. S.; Saugstad J. A.; Shekari F.; Swift S.; Ter-Ovanesyan D.; Tosar J. P.; Useckaite Z.; Valle F.; Varga Z.; van der Pol E.; van Herwijnen M. J. C.; Wauben M. H. M.; Wehman A. M.; Williams S.; Zendrini A.; Zimmerman A. J.; Théry C.; Witwer K. W. Minimal Information for Studies of Extracellular Vesicles (MISEV2023): From Basic to Advanced Approaches. J. Extracell. Vesicles 2024, 13 (2), e12404 10.1002/jev2.12404. 38326288 PMC10850029

[ref23] Bebelman M. P.; Smit M. J.; Pegtel D. M.; Baglio S. R. Biogenesis and Function of Extracellular Vesicles in Cancer. Pharmacol. Ther. 2018, 188, 1 10.1016/j.pharmthera.2018.02.013. 29476772

[ref24] Saleem T.; Sumrin A.; Bilal M.; Bashir H.; Khawar M. B. Tumor-Derived Extracellular Vesicles: Potential Tool for Cancer Diagnosis, Prognosis, and Therapy. Saudi J. Biol. Sci. 2022, 29 (4), 2063–2071. 10.1016/j.sjbs.2022.01.012. 35531155 PMC9073005

[ref25] Di Vizio D.; Morello M.; Dudley A. C.; Schow P. W.; Adam R. M.; Morley S.; Mulholland D.; Rotinen M.; Hager M. H.; Insabato L.; Moses M. A.; Demichelis F.; Lisanti M. P.; Wu H.; Klagsbrun M.; Bhowmick N. A.; Rubin M. A.; D’Souza-Schorey C.; Freeman M. R. Large Oncosomes in Human Prostate Cancer Tissues and in the Circulation of Mice with Metastatic Disease. Am. J. Pathol. 2012, 181 (5), 1573–1584. 10.1016/j.ajpath.2012.07.030. 23022210 PMC3483805

[ref26] Yang Q.; Xu J.; Gu J.; Shi H.; Zhang J.; Zhang J.; Chen Z. S.; Fang X.; Zhu T.; Zhang X. Extracellular Vesicles in Cancer Drug Resistance: Roles, Mechanisms, and Implications. Adv. Sci. 2022, 9 (34), 2201609 10.1002/advs.202201609. PMC9731723 36253096

[ref27] Chen H.; Wang L.; Zeng X.; Schwarz H.; Nanda H. S.; Peng X.; Zhou Y. Exosomes, a New Star for Targeted Delivery. Front. Cell Dev. Biol. 2021, 9, 751079 10.3389/fcell.2021.751079. 34692704 PMC8531489

[ref28] Thangaraju K.; Neerukonda S. N.; Katneni U.; Buehler P. W. Extracellular Vesicles from Red Blood Cells and Their Evolving Roles in Health, Coagulopathy and Therapy. Int. J. Mol. Sci. 2021, 22 (1), 153 10.3390/ijms22010153. PMC7796437 33375718

[ref29] Alenquer M.; Amorim M. J. Exosome Biogenesis, Regulation, and Function in Viral Infection. Viruses 2015, 7 (9), 5066–5083. 10.3390/v7092862. 26393640 PMC4584306

[ref30] Battistelli M.; Falcieri E. Apoptotic Bodies: Particular Extracellular Vesicles Involved in Intercellular Communication. Biology (Basel). 2020, 9 (1), 21 10.3390/biology9010021. 31968627 PMC7168913

[ref31] Valadi H.; Ekström K.; Bossios A.; Sjöstrand M.; Lee J. J.; Lötvall J. O. Exosome-Mediated Transfer of MRNAs and MicroRNAs Is a Novel Mechanism of Genetic Exchange between Cells. Nat. Cell Biol. 2007 96 2007, 9 (6), 654–659. 10.1038/ncb1596. 17486113

[ref32] Da Costa V. R.; Araldi R. P.; Vigerelli H.; D’Ámelio F.; Mendes T. B.; Gonzaga V.; Policíquio B.; Colozza-Gama G. A.; Valverde C. W.; Kerkis I. Exosomes in the Tumor Microenvironment: From Biology to Clinical Applications. Cells 2021, 10 (10), 2617 10.3390/cells10102617. 34685596 PMC8533895

[ref33] Zaborowski M. P.; Balaj L.; Breakefield X. O.; Lai C. P. Extracellular Vesicles: Composition, Biological Relevance, and Methods of Study. Bioscience 2015, 65 (8), 783–797. 10.1093/biosci/biv084. 26955082 PMC4776721

[ref34] Minciacchi V. R.; Freeman M. R.; Di Vizio D. Extracellular Vesicles in Cancer: Exosomes, Microvesicles and the Emerging Role of Large Oncosomes. Semin. Cell Dev. Biol. 2015, 40, 41–51. 10.1016/j.semcdb.2015.02.010. 25721812 PMC4747631

[ref35] Ståhl A.; Johansson K.; Mossberg M.; Kahn R.; Karpman D. Exosomes and Microvesicles in Normal Physiology, Pathophysiology, and Renal Diseases. Pediatr. Nephrol. 2019, 34 (1), 11 10.1007/s00467-017-3816-z. 29181712 PMC6244861

[ref36] Kamińska K.; Szczylik C.; Bielecka Z. F.; Bartnik E.; Porta C.; Lian F.; Czarnecka A. M. The Role of the Cell-Cell Interactions in Cancer Progression. J. Cell. Mol. Med. 2015, 19 (2), 283–296. 10.1111/jcmm.12408. 25598217 PMC4407603

[ref37] Bayraktar R.; Van Roosbroeck K.; Calin G. A. Cell-to-Cell Communication: MicroRNAs as Hormones. Mol. Oncol. 2017, 11 (12), 1673–1686. 10.1002/1878-0261.12144. 29024380 PMC5709614

[ref38] Crescitelli R.; Lässer C.; Szabó T. G.; Kittel A.; Eldh M.; Dianzani I.; Buzás E. I.; Lötvall J. Distinct RNA Profiles in Subpopulations of Extracellular Vesicles: Apoptotic Bodies, Microvesicles and Exosomes. J. Extracell. Vesicles 2013, 2 (1), 20677 10.3402/jev.v2i0.20677. PMC3823106 24223256

[ref39] Poupardin R.; Wolf M.; Maeding N.; Paniushkina L.; Geissler S.; Bergese P.; Witwer K. W.; Schallmoser K.; Fuhrmann G.; Strunk D. Advances in Extracellular Vesicle Research Over The Past Decade: Source And Isolation Method Are Connected with Cargo And Function. Adv. Healthc. Mater. 2024, 13, 2303941 10.1002/adhm.202303941. 38270559

[ref40] Shirejini S. Z.; Inci F. The Yin and Yang of Exosome Isolation Methods: Conventional Practice, Microfluidics, and Commercial Kits. Biotechnol. Adv. 2022, 54, 107814 10.1016/j.biotechadv.2021.107814. 34389465

[ref41] Sidhom K.; Obi P. O.; Saleem A. A Review of Exosomal Isolation Methods: Is Size Exclusion Chromatography the Best Option?. Int. J. Mol. Sci. 2020, 21 (18), 6466 10.3390/ijms21186466. 32899828 PMC7556044

[ref42] Livshits M. A.; Khomyakova E.; Evtushenko E. G.; Lazarev V. N.; Kulemin N. A.; Semina S. E.; Generozov E. V.; Govorun V. M. Isolation of Exosomes by Differential Centrifugation: Theoretical Analysis of a Commonly Used Protocol. Sci. Rep. 2015, 5 (1), 17319 10.1038/srep17319. 26616523 PMC4663484

[ref43] Li P.; Kaslan M.; Lee S. H.; Yao J.; Gao Z. Progress in Exosome Isolation Techniques. Theranostics 2017, 7 (3), 789 10.7150/thno.18133. 28255367 PMC5327650

[ref44] Cheruvanky A.; Zhou H.; Pisitkun T.; Kopp J. B.; Knepper M. A.; Yuen P. S. T.; Star R. A. Rapid Isolation of Urinary Exosomal Biomarkers Using a Nanomembrane Ultrafiltration Concentrator. Am. J. Physiol. - Ren. Physiol. 2007, 292 (5), F1657–F1661. 10.1152/ajprenal.00434.2006. PMC2271070 17229675

[ref45] Lobb R. J.; Becker M.; Wen S. W.; Wong C. S. F.; Wiegmans A. P.; Leimgruber A.; Möller A. Optimized Exosome Isolation Protocol for Cell Culture Supernatant and Human Plasma. J. Extracell. Vesicles 2015, 4 (1), 27031 10.3402/jev.v4.27031. 26194179 PMC4507751

[ref46] Konoshenko M. Y.; Lekchnov E. A.; Vlassov A. V.; Laktionov P. P. Isolation of Extracellular Vesicles: General Methodologies and Latest Trends. Biomed. Res. Int. 2018, 2018, 1 10.1155/2018/8545347. PMC5831698 29662902

[ref47] Boriachek K.; Masud M. K.; Palma C.; Phan H. P.; Yamauchi Y.; Hossain M. S. A.; Nguyen N. T.; Salomon C.; Shiddiky M. J. A. Avoiding Pre-Isolation Step in Exosome Analysis: Direct Isolation and Sensitive Detection of Exosomes Using Gold-Loaded Nanoporous Ferric Oxide Nanozymes. Anal. Chem. 2019, 91 (6), 3827–3834. 10.1021/acs.analchem.8b03619. 30735354

[ref48] Koliha N.; Wiencek Y.; Heider U.; Jüngst C.; Kladt N.; Krauthäuser S.; Johnston I. C. D.; Bosio A.; Schauss A.; Wild S. A Novel Multiplex Bead-Based Platform Highlights the Diversity of Extracellular Vesicles. J. Extracell. Vesicles 2016, 5 (1), 29975 10.3402/jev.v5.29975. 26901056 PMC4762227

[ref49] Böing A. N.; van der Pol E.; Grootemaat A. E.; Coumans F. A. W.; Sturk A.; Nieuwland R. Single-Step Isolation of Extracellular Vesicles by Size-Exclusion Chromatography. J. Extracell. Vesicles 2014, 3 (1), 23430 10.3402/jev.v3.23430. PMC4159761 25279113

[ref50] Inci F. Benchmarking a Microfluidic-Based Filtration for Isolating Biological Particles. Langmuir 2022, 38 (5), 1897–1909. 10.1021/acs.langmuir.1c03119. 35041413

[ref51] Liang L.-G.; Sheng Y.-F.; Zhou S.; Inci F.; Li L.; Demirci U.; Wang S. An Integrated Double-Filtration Microfluidic Device for Detection of Extracellular Vesicles from Urine for Bladder Cancer Diagnosis. In Extracellular Vesicles: Methods and Protocols; Kuo W. P., Jia S., Eds.; Springer New York: New York, NY, 2017; pp 355–364. 10.1007/978-1-4939-7253-1_29. 28828671

[ref52] Chen C.; Skog J.; Hsu C. H.; Lessard R. T.; Balaj L.; Wurdinger T.; Carter B. S.; Breakefield X. O.; Toner M.; Irimia D. Microfluidic Isolation and Transcriptome Analysis of Serum Microvesicles. Lab Chip 2010, 10 (4), 505–511. 10.1039/B916199F. 20126692 PMC3136803

[ref53] Shehzad A.; Islam S. U.; Shahzad R.; Khan S.; Lee Y. S. Extracellular Vesicles in Cancer Diagnostics and Therapeutics. Pharmacol. Ther. 2021, 223, 107806 10.1016/j.pharmthera.2021.107806. 33465400

[ref54] Chen Y.; Xie Y.; Xu L.; Zhan S.; Xiao Y.; Gao Y.; Wu B.; Ge W. Protein Content and Functional Characteristics of Serum-Purified Exosomes from Patients with Colorectal Cancer Revealed by Quantitative Proteomics. Int. J. Cancer 2017, 140 (4), 900–913. 10.1002/ijc.30496. 27813080

[ref55] Raab-Traub N.; Dittmer D. P. Viral Effects on the Content and Function of Extracellular Vesicles. Nat. Rev. Microbiol. 2017 159 2017, 15 (9), 559–572. 10.1038/nrmicro.2017.60. PMC5555775 28649136

[ref56] Yang S.; Che S. P. Y.; Kurywchak P.; Tavormina J. L.; Gansmo L. B.; Correa de Sampaio P.; Tachezy M.; Bockhorn M.; Gebauer F.; Haltom A. R.; Melo S. A.; LeBleu V. S.; Kalluri R. Detection of Mutant KRAS and TP53 DNA in Circulating Exosomes from Healthy Individuals and Patients with Pancreatic Cancer. Cancer Biol. Ther. 2017, 18 (3), 158–165. 10.1080/15384047.2017.1281499. 28121262 PMC5389423

[ref57] Chen G.; Huang A. C.; Zhang W.; Zhang G.; Wu M.; Xu W.; Yu Z.; Yang J.; Wang B.; Sun H.; Xia H.; Man Q.; Zhong W.; Antelo L. F.; Wu B.; Xiong X.; Liu X.; Guan L.; Li T.; Liu S.; Yang R.; Lu Y.; Dong L.; McGettigan S.; Somasundaram R.; Radhakrishnan R.; Mills G.; Lu Y.; Kim J.; Chen Y. H.; Dong H.; Zhao Y.; Karakousis G. C.; Mitchell T. C.; Schuchter L. M.; Herlyn M.; Wherry E. J.; Xu X.; Guo W. Exosomal PD-L1 Contributes to Immunosuppression and Is Associated with Anti-PD-1 Response. Nature 2018, 560 (7718), 382–386. 10.1038/s41586-018-0392-8. 30089911 PMC6095740

[ref58] Hoshino A.; Costa-Silva B.; Shen T. L.; Rodrigues G.; Hashimoto A.; Tesic Mark M.; Molina H.; Kohsaka S.; Di Giannatale A.; Ceder S.; Singh S.; Williams C.; Soplop N.; Uryu K.; Pharmer L.; King T.; Bojmar L.; Davies A. E.; Ararso Y.; Zhang T.; Zhang H.; Hernandez J.; Weiss J. M.; Dumont-Cole V. D.; Kramer K.; Wexler L. H.; Narendran A.; Schwartz G. K.; Healey J. H.; Sandstrom P.; Jørgen Labori K.; Kure E. H.; Grandgenett P. M.; Hollingsworth M. A.; De Sousa M.; Kaur S.; Jain M.; Mallya K.; Batra S. K.; Jarnagin W. R.; Brady M. S.; Fodstad O.; Muller V.; Pantel K.; Minn A. J.; Bissell M. J.; Garcia B. A.; Kang Y.; Rajasekhar V. K.; Ghajar C. M.; Matei I.; Peinado H.; Bromberg J.; Lyden D. Tumour Exosome Integrins Determine Organotropic Metastasis. Nature 2015, 527 (7578), 329–335. 10.1038/nature15756. 26524530 PMC4788391

[ref59] Dong L.; Pu Y.; Zhang L.; Qi Q.; Xu L.; Li W.; Wei C.; Wang X.; Zhou S.; Zhu J.; Wang X.; Liu F.; Chen X.; Su C. Human Umbilical Cord Mesenchymal Stem Cell-Derived Extracellular Vesicles Promote Lung Adenocarcinoma Growth by Transferring MiR-410. Cell Death Dis. 2018, 9 (2), 1–13. 10.1038/s41419-018-0323-5. 29440630 PMC5833395

[ref60] Dong H.; Wang W.; Chen R.; Zhang Y.; Zou K.; Ye M.; He X.; Zhang F.; Han J. Exosome-Mediated Transfer of LncRNA-SNHG14 Promotes Trastuzumab Chemoresistance in Breast Cancer. Int. J. Oncol. 2018, 53 (3), 1013–1026. 10.3892/ijo.2018.4467. 30015837 PMC6065402

[ref61] Cao Y. L.; Zhuang T.; Xing B. H.; Li N.; Li Q. Exosomal DNMT1 Mediates Cisplatin Resistance in Ovarian Cancer. Cell Biochem. Funct. 2017, 35 (6), 296–303. 10.1002/cbf.3276. 28791708

[ref62] Liu J.; Jalali M.; Mahshid S.; Wachsmann-Hogiu S. Are Plasmonic Optical Biosensors Ready for Use in Point-of-Need Applications?. Analyst 2020, 145 (2), 364–384. 10.1039/C9AN02149C. 31832630

[ref63] Uniyal A.; Srivastava G.; Pal A.; Taya S.; Muduli A. Recent Advances in Optical Biosensors for Sensing Applications: A Review. Plasmonics 2023, 18 (2), 735–750. 10.1007/s11468-023-01803-2.

[ref64] Soler M.; Lechuga L. M. Principles, Technologies, and Applications of Plasmonic Biosensors. J. Appl. Phys. 2021, 129 (11), 111102 10.1063/5.0042811.

[ref65] Ahmed R.; Ozen M. O.; Karaaslan M. G.; Prator C. A.; Thanh C.; Kumar S.; Torres L.; Iyer N.; Munter S.; Southern S.; Henrich T. J.; Inci F.; Demirci U. Tunable Fano-Resonant Metasurfaces on a Disposable Plastic-Template for Multimodal and Multiplex Biosensing. Adv. Mater. 2020, 32 (19), e1907160 10.1002/adma.201907160. 32201997 PMC8713081

[ref66] Wood R. W. On a Remarkable Case of Uneven Distribution of Light in a Diffraction Grating Spectrum. Proc. Phys. Soc. London 1901, 18 (1), 269 10.1088/1478-7814/18/1/325.

[ref67] Fano U. The Theory of Anomalous Diffraction Gratings and of Quasi-Stationary Waves on Metallic Surfaces (Sommerfeld’s Waves). J. Opt. Soc. Am. 1941, 31 (3), 213–222. 10.1364/JOSA.31.000213.

[ref68] Liedberg B.; Nylander C.; Lundström I. Biosensing with Surface Plasmon Resonance — How It All Started. Biosens. Bioelectron. 1995, 10 (8), i–ix. 10.1016/0956-5663(95)96965-2. 7576432

[ref69] Kowalczyk A.; Gajda-Walczak A.; Ruzycka-Ayoush M.; Targonska A.; Mosieniak G.; Glogowski M.; Szumera-Cieckiewicz A.; Prochorec-Sobieszek M.; Bamburowicz-Klimkowska M.; Nowicka A. M.; Grudzinski I. P. Parallel SPR and QCM-D Quantitative Analysis of CD9, CD63, and CD81 Tetraspanins: A Simple and Sensitive Way to Determine the Concentration of Extracellular Vesicles Isolated from Human Lung Cancer Cells. Anal. Chem. 2023, 95 (25), 9520–9530. 10.1021/acs.analchem.3c00772. 37307147 PMC10308334

[ref70] Wang Q.; Zou L.; Yang X.; Liu X.; Nie W.; Zheng Y.; Cheng Q.; Wang K. Direct Quantification of Cancerous Exosomes via Surface Plasmon Resonance with Dual Gold Nanoparticle-Assisted Signal Amplification. Biosens. Bioelectron. 2019, 135, 129–136. 10.1016/j.bios.2019.04.013. 31004923

[ref71] Liu C.; Zeng X.; An Z.; Yang Y.; Eisenbaum M.; Gu X.; Jornet J. M.; Dy G. K.; Reid M. E.; Gan Q.; Wu Y. Sensitive Detection of Exosomal Proteins via a Compact Surface Plasmon Resonance Biosensor for Cancer Diagnosis. ACS Sensors 2018, 3 (8), 1471–1479. 10.1021/acssensors.8b00230. 30019892 PMC8628517

[ref72] Mayer K. M.; Hafner J. H. Localized Surface Plasmon Resonance Sensors. Chem. Rev. 2011, 111 (6), 3828–3857. 10.1021/cr100313v. 21648956

[ref73] Sui M.; Kunwar S.; Pandey P.; Lee J. Strongly Confined Localized Surface Plasmon Resonance (LSPR) Bands of Pt, AgPt, AgAuPt Nanoparticles. Sci. Rep. 2019, 9 (1), 16582 10.1038/s41598-019-53292-1. 31719664 PMC6851101

[ref74] Li P.; Long F.; Chen W.; Chen J.; Chu P. K.; Wang H. Fundamentals and Applications of Surface-Enhanced Raman Spectroscopy-based Biosensors. Curr. Opin. Biomed. Eng. 2020, 13, 51–59. 10.1016/j.cobme.2019.08.008.

[ref75] Chauhan P.; Bhargava A.; Kumari R.; Ratre P.; Tiwari R.; Kumar Srivastava R.; Goryacheva I. Yu.; Kumar Mishra P. Surface-Enhanced Raman Scattering Biosensors for Detection of OncomiRs in Breast Cancer. Drug Discovery Today 2022, 27 (8), 2121–2136. 10.1016/j.drudis.2022.04.016. 35460892

[ref76] Zheng H.; Ding Q.; Li C.; Chen W.; Chen X.; Lin Q.; Wang D.; Weng Y.; Lin D. Recent Progress in Surface-Enhanced Raman Spectroscopy-Based Biosensors for the Detection of Extracellular Vesicles. Anal. Methods 2022, 14 (42), 4161–4173. 10.1039/D2AY01339H. 36254847

[ref77] Li J.; Li Y.; Li P.; Zhang Y.; Du L.; Wang Y.; Zhang C.; Wang C. Exosome Detection via Surface-Enhanced Raman Spectroscopy for Cancer Diagnosis. Acta Biomater. 2022, 144, 1–14. 10.1016/j.actbio.2022.03.036. 35358734

[ref78] Shao B.; Xiao Z. Recent Achievements in Exosomal Biomarkers Detection by Nanomaterials-Based Optical Biosensors - A Review. Anal. Chim. Acta 2020, 1114, 74–84. 10.1016/j.aca.2020.02.041. 32359518

[ref79] Sahl S. J.; Hell S. W.; Jakobs S. Fluorescence Nanoscopy in Cell Biology. Nat. Rev. Mol. Cell Biol. 2017, 18 (11), 685–701. 10.1038/nrm.2017.71. 28875992

[ref80] Brahmkhatri V.; Pandit P.; Rananaware P.; D’Souza A.; Kurkuri M. D. Recent Progress in Detection of Chemical and Biological Toxins in Water Using Plasmonic Nanosensors. Trends Environ. Anal. Chem. 2021, 30, e00117 10.1016/j.teac.2021.e00117.

[ref81] Zhu N.; Li G.; Zhou J.; Zhang Y.; Kang K.; Ying B.; Yi Q.; Wu Y. A Light-up Fluorescence Resonance Energy Transfer Magnetic Aptamer-Sensor for Ultra-Sensitive Lung Cancer Exosome Detection. J. Mater. Chem. B 2021, 9 (10), 2483–2493. 10.1039/D1TB00046B. 33656037

[ref82] Thorsteinsson K.; Olsén E.; Schmidt E.; Pace H.; Bally M. FRET-Based Assay for the Quantification of Extracellular Vesicles and Other Vesicles of Complex Composition. Anal. Chem. 2020, 92 (23), 15336–15343. 10.1021/acs.analchem.0c02271. PMC7735656 33179908

[ref83] Fu X.; Song Y.; Masud A.; Nuti K.; DeRouchey J. E.; Richards C. I. High-Throughput Fluorescence Correlation Spectroscopy Enables Analysis of Surface Components of Cell-Derived Vesicles. Anal. Bioanal. Chem. 2020, 412 (11), 2589–2597. 10.1007/s00216-020-02485-z. PMC8492160 32146499

[ref84] Wyss R.; Grasso L.; Wolf C.; Grosse W.; Demurtas D.; Vogel H. Molecular and Dimensional Profiling of Highly Purified Extracellular Vesicles by Fluorescence Fluctuation Spectroscopy. Anal. Chem. 2014, 86 (15), 7229–7233. 10.1021/ac501801m. 25001505

[ref85] Manzano M. Chapter 6 - Labelled and Unlabelled Probes for Pathogen Detection with Molecular Biology Methods and Biosensors. In Fluorescent Probes; Gurtler V., Ed.; Methods in Microbiology 48; Academic Press: 2021; pp 179–225. 10.1016/bs.mim.2021.03.001.

[ref86] Zeni L.; Perri C.; Cennamo N.; Arcadio F.; D’Agostino G.; Salmona M.; Beeg M.; Gobbi M. A Portable Optical-Fibre-Based Surface Plasmon Resonance Biosensor for the Detection of Therapeutic Antibodies in Human Serum. Sci. Rep. 2020, 10 (1), 11154 10.1038/s41598-020-68050-x. PMC7341820 32636434

[ref87] Yildizhan Y.; Driessens K.; Tsao H. S. K.; Boiy R.; Thomas D.; Geukens N.; Hendrix A.; Lammertyn J.; Spasic D. Detection of Breast Cancer-Specific Extracellular Vesicles with Fiber-Optic SPR Biosensor. Int. J. Mol. Sci. 2023, 24 (4), 3764 10.3390/ijms24043764. PMC9966403 36835174

[ref88] Li S.; Zhu L.; Zhu L.; Mei X.; Xu W. A Sandwich-Based Evanescent Wave Fluorescent Biosensor for Simple, Real-Time Exosome Detection†. Biosens. Bioelectron. 2022, 200, 113902 10.1016/j.bios.2021.113902. 34954570

[ref89] Kaur B.; Kumar S.; Kaushik B. K. Recent Advancements in Optical Biosensors for Cancer Detection. Biosens. Bioelectron. 2022, 197, 113805 10.1016/j.bios.2021.113805. 34801795

[ref90] Xia Z.; Zhang X.; Yao J.; Liu Z.; Jin Y.; Yin H.; Wang P.; Wang X.-H. Giant Enhancement of Raman Scattering by a Hollow-Core Microstructured Optical Fiber Allows Single Exosome Probing. ACS Sensors 2023, 8 (4), 1799–1809. 10.1021/acssensors.3c00131. 37018734

[ref91] Chiavaioli F.; Baldini F.; Tombelli S.; Trono C.; Giannetti A. Biosensing with Optical Fiber Gratings. Nanophotonics 2017, 6 (4), 663–679. 10.1515/nanoph-2016-0178.

[ref92] Chen S.; Zhang C.; Wang J.; Li N.; Song Y.; Wu H.; Liu Y. A Fiber Bragg Grating Sensor Based on Cladding Mode Resonance for Label-Free Biosensing. Biosensors 2023, 13 (1), 97 10.3390/bios13010097. 36671932 PMC9855977

[ref93] Quero G.; Consales M.; Severino R.; Vaiano P.; Boniello A.; Sandomenico A.; Ruvo M.; Borriello A.; Diodato L.; Zuppolini S.; Giordano M.; Nettore I. C.; Mazzarella C.; Colao A.; Macchia P. E.; Santorelli F.; Cutolo A.; Cusano A. Long Period Fiber Grating Nano-Optrode for Cancer Biomarker Detection. Biosens. Bioelectron. 2016, 80, 590–600. 10.1016/j.bios.2016.02.021. 26896794

[ref94] Lou U. K.; Wong C. H.; Chen Y. A Simple and Rapid Colorimetric Detection of Serum LncRNA Biomarkers for Diagnosis of Pancreatic Cancer. RSC Adv. 2020, 10 (14), 8087–8092. 10.1039/C9RA07858D. 35497850 PMC9049936

[ref95] Liu M.-X.; Zhang H.; Chen S.; Yu Y.-L.; Wang J.-H. MnO2-Graphene Oxide Hybrid Nanomaterial with Oxidase-like Activity for Ultrasensitive Colorimetric Detection of Cancer Cells. Anal. Bioanal. Chem. 2021, 413 (17), 4451–4458. 10.1007/s00216-021-03399-0. 34002276

[ref96] Xu L.; Chopdat R.; Li D.; Al-Jamal K. T. Development of a Simple, Sensitive and Selective Colorimetric Aptasensor for the Detection of Cancer-Derived Exosomes. Biosens. Bioelectron. 2020, 169, 112576 10.1016/j.bios.2020.112576. 32919211

[ref97] Zhang Y.; Jiao J.; Wei Y.; Wang D.; Yang C.; Xu Z. Plasmonic Colorimetric Biosensor for Sensitive Exosome Detection via Enzyme-Induced Etching of Gold Nanobipyramid@MnO2 Nanosheet Nanostructures. Anal. Chem. 2020, 92 (22), 15244–15252. 10.1021/acs.analchem.0c04136. 33108733

[ref98] Fattahi Z.; Khosroushahi A. Y.; Hasanzadeh M. Recent Progress on Developing of Plasmon Biosensing of Tumor Biomarkers: Efficient Method towards Early Stage Recognition of Cancer. Biomed. Pharmacother. 2020, 132, 110850 10.1016/j.biopha.2020.110850. 33068930

[ref99] Ouyang N.; Hong L.; Zhou Y.; Zhang J.; Shafi S.; Pan J.; Zhao R.; Yang Y.; Hou W. Application of Fluorescent Nano-Biosensor for the Detection of Cancer Bio-Macromolecular Markers. Polym. Test. 2022, 115, 107746 10.1016/j.polymertesting.2022.107746.

[ref100] Farhana F. Z.; Umer M.; Saeed A.; Pannu A. S.; Shahbazi M.; Jabur A.; Nam H. J.; Ostrikov K.; Sonar P.; Firoz S. H.; Shiddiky M. J. A. Isolation and Detection of Exosomes Using Fe2O3 Nanoparticles. ACS Appl. Nano Mater. 2021, 4 (2), 1175–1186. 10.1021/acsanm.0c02807.

[ref101] Liu S. Y.; Liao Y.; Hosseinifard H.; Imani S.; Wen Q. L. Diagnostic Role of Extracellular Vesicles in Cancer: A Comprehensive Systematic Review and Meta-Analysis. Front. Cell Dev. Biol. 2021, 9, 705791 10.3389/fcell.2021.705791. 34722499 PMC8555429

[ref102] Ju R. J.; Li X. T.; Shi J. F.; Li X. Y.; Sun M. G.; Zeng F.; Zhou J.; Liu L.; Zhang C. X.; Zhao W. Y.; Lu W. L. Liposomes, Modified with PTDHIV-1 Peptide, Containing Epirubicin and Celecoxib, to Target Vasculogenic Mimicry Channels in Invasive Breast Cancer. Biomaterials 2014, 35 (26), 7610–7621. 10.1016/j.biomaterials.2014.05.040. 24912818

[ref103] Marrugo-Ramírez J.; Mir M.; Samitier J. Blood-Based Cancer Biomarkers in Liquid Biopsy: A Promising Non-Invasive Alternative to Tissue Biopsy. Int. J. Mol. Sci. 2018, Vol. 19, Page 2877 2018, 19 (10), 2877 10.3390/ijms19102877. PMC6213360 30248975

[ref104] Ferguson S.; Yang K. S.; Weissleder R. Single Extracellular Vesicle Analysis for Early Cancer Detection. Trends Mol. Med. 2022, 28, 681 10.1016/j.molmed.2022.05.003. 35624008 PMC9339504

[ref105] Kosaka N.; Kogure A.; Yamamoto T.; Urabe F.; Usuba W.; Prieto-Vila M.; Ochiya T. Exploiting the Message from Cancer: The Diagnostic Value of Extracellular Vesicles for Clinical Applications. Exp. Mol. Med. 2019, 51 (3), 1–9. 10.1038/s12276-019-0219-1. PMC6418231 30872565

[ref106] Ma C.; Jiang F.; Ma Y.; Wang J.; Li H.; Zhang J. Isolation and Detection Technologies of Extracellular Vesicles and Application on Cancer Diagnostic. Dose-Response 2019, 10.1177/1559325819891004. PMC6902397 31839757

[ref107] George S. K.; Lauková L.; Weiss R.; Semak V.; Fendl B.; Weiss V. U.; Steinberger S.; Allmaier G.; Tripisciano C.; Weber V. Comparative Analysis of Platelet-Derived Extracellular Vesicles Using Flow Cytometry and Nanoparticle Tracking Analysis. Int. J. Mol. Sci. 2021, 22 (8), 3839 10.3390/ijms22083839. 33917210 PMC8068037

[ref108] Jurgielewicz B. J.; Yao Y.; Stice S. L. Kinetics and Specificity of HEK293T Extracellular Vesicle Uptake Using Imaging Flow Cytometry. Nanoscale Res. Lett. 2020, 15, 170 10.1186/s11671-020-03399-6. 32833066 PMC7445225

[ref109] Logozzi M.; Di Raimo R.; Mizzoni D.; Fais S. Immunocapture-Based ELISA to Characterize and Quantify Exosomes in Both Cell Culture Supernatants and Body Fluids. Methods Enzymol. 2020, 645, 155–180. 10.1016/bs.mie.2020.06.011. 33565970 PMC7346819

[ref110] Kim J. S.; Kwon S. Y.; Lee J. Y.; Kim S. D.; Kim H.; Jang N.; Wang J.; Han M.; Kong S. H.; Kim D. Y. High-Throughput Multi-Gate Microfluidic Resistive Pulse Sensing for Biological Nanoparticle Detection. Lab Chip 2023, 23 (7), 1945–1953. 10.1039/D2LC01064J. 36897079

[ref111] Young T. W.; Kappler M. P.; Hockaden N. M.; Carpenter R. L.; Jacobson S. C. Characterization of Extracellular Vesicles by Resistive-Pulse Sensing on In-Plane Multipore Nanofluidic Devices. Anal. Chem. 2023, 95, 16710 10.1021/acs.analchem.3c03546. 37916500 PMC10841850

[ref112] Lyu T. S.; Ahn Y.; Im Y.-J.; Kim S.-S.; Lee K.-H.; Kim J.; Choi Y.; Lee D.; Kang E.; Jin G.; et al. The Characterization of Exosomes from Fibrosarcoma Cell and the Useful Usage of Dynamic Light Scattering (DLS) for Their Evaluation. PLoS One 2021, 16 (1), e0231994 10.1371/journal.pone.0231994. 33497388 PMC7837462

[ref113] Serrano-Pertierra E.; Oliveira-Rodríguez M.; Rivas M.; Oliva P.; Villafani J.; Navarro A.; Blanco-López M. C.; Cernuda-Morollón E. Characterization of Plasma-Derived Extracellular Vesicles Isolated by Different Methods: A Comparison Study. Bioengineering 2019, 6 (1), 8 10.3390/bioengineering6010008. 30658418 PMC6466225

[ref114] Droste M.; Tertel T.; Jeruschke S.; Dittrich R.; Kontopoulou E.; Walkenfort B.; Börger V.; Hoyer P. F.; Büscher A. K.; Thakur B. K.; Giebel B. Single Extracellular Vesicle Analysis Performed by Imaging Flow Cytometry and Nanoparticle Tracking Analysis Evaluate the Accuracy of Urinary Extracellular Vesicle Preparation Techniques Differently. Int. J. Mol. Sci. 2021, 22 (22), 12436 10.3390/ijms222212436. 34830318 PMC8620260

[ref115] Tan K. L.; Chia W. C.; How C. W.; Tor Y. S.; Show P. L.; Looi Q. H. D.; Foo J. B. Benchtop Isolation and Characterisation of Small Extracellular Vesicles from Human Mesenchymal Stem Cells. Mol. Biotechnol. 2021, 63 (9), 780–791. 10.1007/s12033-021-00339-2. 34061307

[ref116] Tulkens J.; De Wever O.; Hendrix A. Analyzing Bacterial Extracellular Vesicles in Human Body Fluids by Orthogonal Biophysical Separation and Biochemical Characterization. Nat. Protoc. 2020, 15 (1), 40–67. 10.1038/s41596-019-0236-5. 31776460

[ref117] Liang Y.; Huang S.; Qiao L.; Peng X.; Li C.; Lin K.; Xie G.; Li J.; Lin L.; Yin Y.; et al. Characterization of Protein, Long Noncoding RNA and MicroRNA Signatures in Extracellular Vesicles Derived from Resting and Degranulated Mast Cells. J. Extracell. Vesicles 2020, 9 (1), 1697583 10.1080/20013078.2019.1697583. 31853339 PMC6913652

[ref118] Burrello J.; Gai C.; Tetti M.; Lopatina T.; Deregibus M. C.; Veglio F.; Mulatero P.; Camussi G.; Monticone S. Characterization and Gene Expression Analysis of Serum-Derived Extracellular Vesicles in Primary Aldosteronism. Hypertension 2019, 74 (2), 359–367. 10.1161/HYPERTENSIONAHA.119.12944. 31230554

[ref119] Bari S. M. I.; Hossain F. B.; Nestorova G. G. Advances in Biosensors Technology for Detection and Characterization of Extracellular Vesicles. Sensors 2021, 21 (22), 7645 10.3390/s21227645. 34833721 PMC8621354

[ref120] Wang S. U.; Khan A.; Huang R.; Ye S.; Di K.; Xiong T.; Li Z. Recent Advances in Single Extracellular Vesicle Detection Methods. Biosens. Bioelectron. 2020, 154, 112056 10.1016/j.bios.2020.112056. 32093894

[ref121] Jahani Y.; Arvelo E. R.; Yesilkoy F.; Koshelev K.; Cianciaruso C.; De Palma M.; Kivshar Y.; Altug H. Imaging-Based Spectrometer-Less Optofluidic Biosensors Based on Dielectric Metasurfaces for Detecting Extracellular Vesicles. Nat. Commun. 2021, 12 (1), 3246 10.1038/s41467-021-23257-y. 34059690 PMC8167130

[ref122] Kilic T.; Valinhas A. T. D. S.; Wall I.; Renaud P.; Carrara S. Label-Free Detection of Hypoxia-Induced Extracellular Vesicle Secretion from MCF-7 Cells. Sci. Rep. 2018, 8 (1), 9402 10.1038/s41598-018-27203-9. 29925885 PMC6010476

[ref123] Mathew D. G.; Beekman P.; Lemay S. G.; Zuilhof H.; Le Gac S.; van der Wiel W. G. Electrochemical Detection of Tumor-Derived Extracellular Vesicles on Nanointerdigitated Electrodes. Nano Lett. 2020, 20 (2), 820–828. 10.1021/acs.nanolett.9b02741. 31536360 PMC7020140

[ref124] Zhang Y.; Murakami K.; Borra V. J.; Ozen M. O.; Demirci U.; Nakamura T.; Esfandiari L. A Label-Free Electrical Impedance Spectroscopy for Detection of Clusters of Extracellular Vesicles Based on Their Unique Dielectric Properties. Biosensors 2022, 12 (2), 104 10.3390/bios12020104. 35200364 PMC8869858

[ref125] Liu P.; Qian X.; Li X.; Fan L.; Li X.; Cui D.; Yan Y. Enzyme-Free Electrochemical Biosensor Based on Localized DNA Cascade Displacement Reaction and Versatile DNA Nanosheets for Ultrasensitive Detection of Exosomal MicroRNA. ACS Appl. Mater. Interfaces 2020, 12 (40), 45648–45656. 10.1021/acsami.0c14621. 32915531

[ref126] Xu L.; Shoaie N.; Jahanpeyma F.; Zhao J.; Azimzadeh M.; Al-Jamal K. T. Optical, Electrochemical and Electrical (Nano) Biosensors for Detection of Exosomes: A Comprehensive Overview. Biosens. Bioelectron. 2020, 161, 112222 10.1016/j.bios.2020.112222. 32365010

[ref127] Guo F.; Sun M.; Zhang Y.; Xie J.; Gao Q.; Duan W.-J.; Chen J.-X.; Chen J.; Dai Z.; Li M. A Dual Aptamer Recognition-Based Fluorescent Biosensor for Extracellular Vesicles Assays with High Sensitivity and Specificity. Sensors Actuators B Chem. 2023, 389, 133890 10.1016/j.snb.2023.133890.

[ref128] Cheng S.; Kong Q.; Hu X.; Zhang C.; Xian Y. An Ultrasensitive Strand Displacement Signal Amplification-Assisted Synchronous Fluorescence Assay for Surface Proteins of Small Extracellular Vesicle Analysis and Cancer Identification. Anal. Chem. 2022, 94 (2), 1085–1091. 10.1021/acs.analchem.1c04122. 35042294

[ref129] Zhang W.; Jiang L.; Diefenbach R. J.; Campbell D. H.; Walsh B. J.; Packer N. H.; Wang Y. Enabling Sensitive Phenotypic Profiling of Cancer-Derived Small Extracellular Vesicles Using Surface-Enhanced Raman Spectroscopy Nanotags. ACS sensors 2020, 5 (3), 764–771. 10.1021/acssensors.9b02377. 32134252

[ref130] Kwizera E. A.; O’Connor R.; Vinduska V.; Williams M.; Butch E. R.; Snyder S. E.; Chen X.; Huang X. Molecular Detection and Analysis of Exosomes Using Surface-Enhanced Raman Scattering Gold Nanorods and a Miniaturized Device. Theranostics 2018, 8 (10), 2722 10.7150/thno.21358. 29774071 PMC5957005

[ref131] Sina A. A. I.; Vaidyanathan R.; Wuethrich A.; Carrascosa L. G.; Trau M. Label-Free Detection of Exosomes Using a Surface Plasmon Resonance Biosensor. Anal. Bioanal. Chem. 2019, 411, 1311–1318. 10.1007/s00216-019-01608-5. 30719562

[ref132] Hosseinkhani B.; van den Akker N.; D’Haen J.; Gagliardi M.; Struys T.; Lambrichts I.; Waltenberger J.; Nelissen I.; Hooyberghs J.; Molin D. G. M.; Michiels L. Direct Detection of Nano-Scale Extracellular Vesicles Derived from Inflammation-Triggered Endothelial Cells Using Surface Plasmon Resonance. Nanomed.: Nanotechnol., Biol. Med. 2017, 13 (5), 1663–1671. 10.1016/j.nano.2017.03.010. 28366819

[ref133] Xia Y.; Chen T.; Chen G.; Weng Y.; Zeng L.; Liao Y.; Chen W.; Lan J.; Zhang J.; Chen J. A Nature-Inspired Colorimetric and Fluorescent Dual-Modal Biosensor for Exosomes Detection. Talanta 2020, 214, 120851 10.1016/j.talanta.2020.120851. 32278412

[ref134] Lv S. W.; Liu Y.; Xie M.; Wang J.; Yan X. W.; Li Z.; Dong W. G.; Huang W. H. Near-Infrared Light-Responsive Hydrogel for Specific Recognition and Photothermal Site-Release of Circulating Tumor Cells. ACS Nano 2016, 10 (6), 6201–6210. 10.1021/acsnano.6b02208. 27299807

[ref135] Liang Y.; Lehrich B. M.; Zheng S.; Lu M. Emerging Methods in Biomarker Identification for Extracellular Vesicle-based Liquid Biopsy. J. Extracell. Vesicles 2021, 10 (7), e12090 10.1002/jev2.12090. 34012517 PMC8114032

[ref136] Haldavnekar R.; Venkatakrishnan K.; Tan B. Cancer Stem Cell Derived Extracellular Vesicles with Self-Functionalized 3D Nanosensor for Real-Time Cancer Diagnosis: Eliminating the Roadblocks in Liquid Biopsy. ACS Nano 2022, 16 (8), 12226–12243. 10.1021/acsnano.2c02971. 35968931

[ref137] Rojalin T.; Koster H. J.; Liu J.; Mizenko R. R.; Tran D.; Wachsmann-Hogiu S.; Carney R. P. Hybrid Nanoplasmonic Porous Biomaterial Scaffold for Liquid Biopsy Diagnostics Using Extracellular Vesicles. ACS sensors 2020, 5 (9), 2820–2833. 10.1021/acssensors.0c00953. 32935542 PMC7522966

[ref138] Thakur A.; Qiu G.; NG S.-P.; Guan J.; Yue J.; Lee Y.; Wu C.-M. L. Direct Detection of Two Different Tumor-Derived Extracellular Vesicles by SAM-AuNIs LSPR Biosensor. Biosens. Bioelectron. 2017, 94, 400–407. 10.1016/j.bios.2017.03.036. 28324860

[ref139] Liang H.; Wang X.; Li F.; Xie Y.; Shen J.; Wang X.; Huang Y.; Lin S.; Chen J.; Zhang L.; Jiang B.; Xing J.; Zhu J. Label-Free Plasmonic Metasensing of PSA and Exosomes in Serum for Rapid High-Sensitivity Diagnosis of Early Prostate Cancer. Biosens. Bioelectron. 2023, 235, 115380 10.1016/j.bios.2023.115380. 37207584

[ref140] Luo X.; Yan S.; Chen G.; Wang Y.; Zhang X.; Lan J.; Chen J.; Yao X. A Cavity Induced Mode Hybridization Plasmonic Sensor for Portable Detection of Exosomes. Biosens. Bioelectron. 2024, 261, 116492 10.1016/j.bios.2024.116492. 38870828

[ref141] Li H.; Wu H.; Lu L.; Zhang J.; Wang X.; Wang W.; Zheng A.; Liang L.; Sun H.; Zheng J.; Yuan H.; Cao Z.; Yu Q.; Yu B.; Wang H. Optical Microfiber Coated with WS2-Supported Gold Nanobipyramids: Ultrasensitive Detecting Prostate Cancer Extracellular Vesicles in Complex Human Samples. Adv. Opt. Mater. 2024, 12 (5), 2301670 10.1002/adom.202301670.

[ref142] Saha N.; Brunetti G.; Kumar A.; Armenise M. N.; Ciminelli C. Highly Sensitive Refractive Index Sensor Based on Polymer Bragg Grating: A Case Study on Extracellular Vesicles Detection. Biosensors 2022, 12 (6), 415 10.3390/bios12060415. 35735562 PMC9220804

[ref143] Zhang J.; Shi J.; Zhang H.; Zhu Y.; Liu W.; Zhang K.; Zhang Z. Localized Fluorescent Imaging of Multiple Proteins on Individual Extracellular Vesicles Using Rolling Circle Amplification for Cancer Diagnosis. J. Extracell. Vesicles 2020, 10 (1), e12025 10.1002/jev2.12025. 33304477 PMC7710127

[ref144] Xi Y.; Xu P. Global Colorectal Cancer Burden in 2020 and Projections to 2040. Transl. Oncol. 2021, 14 (10), 101174 10.1016/j.tranon.2021.101174. 34243011 PMC8273208

[ref145] Moyano A.; Serrano-Pertierra E.; Duque J. M.; Ramos V.; Teruel-Barandiarán E.; Fernández-Sánchez M. T.; Salvador M.; Martínez-García J. C.; Sánchez L.; García-Flórez L.; et al. Magnetic Lateral Flow Immunoassay for Small Extracellular Vesicles Quantification: Application to Colorectal Cancer Biomarker Detection. Sensors 2021, 21 (11), 3756 10.3390/s21113756. 34071520 PMC8199047

[ref146] Koponen A.; Kerkelä E.; Rojalin T.; Lázaro-Ibáñez E.; Suutari T.; Saari H. O.; Siljander P.; Yliperttula M.; Laitinen S.; Viitala T. Label-Free Characterization and Real-Time Monitoring of Cell Uptake of Extracellular Vesicles. Biosens. Bioelectron. 2020, 168, 112510 10.1016/j.bios.2020.112510. 32877783

[ref147] Bordanaba-Florit G.; Royo F.; Kruglik S. G.; Falcon-Perez J. M. Using Single-Vesicle Technologies to Unravel the Heterogeneity of Extracellular Vesicles. Nat. Protoc. 2021, 16 (7), 3163–3185. 10.1038/s41596-021-00551-z. 34135505

[ref148] Huang G.; Lin G.; Zhu Y.; Duan W.; Jin D. Emerging Technologies for Profiling Extracellular Vesicle Heterogeneity. Lab Chip 2020, 20 (14), 2423–2437. 10.1039/D0LC00431F. 32537618

[ref149] Su X.; Liu X.; Xie Y.; Chen M.; Zheng C.; Zhong H.; Li M. Integrated SERS-Vertical Flow Biosensor Enabling Multiplexed Quantitative Profiling of Serological Exosomal Proteins in Patients for Accurate Breast Cancer Subtyping. ACS Nano 2023, 17 (4), 4077–4088. 10.1021/acsnano.3c00449. 36758150

[ref150] Xie Y.; Su X.; Wen Y.; Zheng C.; Li M. Artificial Intelligent Label-Free SERS Profiling of Serum Exosomes for Breast Cancer Diagnosis and Postoperative Assessment. Nano Lett. 2022, 22 (19), 7910–7918. 10.1021/acs.nanolett.2c02928. 36149810

[ref151] Singh K.; Nalabotala R.; Koo K. M.; Bose S.; Nayak R.; Shiddiky M. J. A. Separation of Distinct Exosome Subpopulations: Isolation and Characterization Approaches and Their Associated Challenges. Analyst 2021, 146 (12), 3731–3749. 10.1039/D1AN00024A. 33988193

[ref152] Parlatan U.; Ozen M. O.; Kecoglu I.; Koyuncu B.; Torun H.; Khalafkhany D.; Loc I.; Ogut M. G.; Inci F.; Akin D.; Solaroglu I.; Ozoren N.; Unlu M. B.; Demirci U. Label-Free Identification of Exosomes Using Raman Spectroscopy and Machine Learning. Small 2023, 19 (9), 2205519 10.1002/smll.202205519. 36642804

[ref153] Petroni D.; Fabbri C.; Babboni S.; Menichetti L.; Basta G.; Del Turco S. Extracellular Vesicles and Intercellular Communication: Challenges for In Vivo Molecular Imaging and Tracking. Pharmaceutics 2023, 15 (6), 1639 10.3390/pharmaceutics15061639. 37376087 PMC10301899

[ref154] Kurt H.; Pishva P.; Pehlivan Z. S.; Arsoy E. G.; Saleem Q.; Bayazıt M. K.; Yüce M. Nanoplasmonic Biosensors: Theory, Structure, Design, and Review of Recent Applications. Anal. Chim. Acta 2021, 1185, 338842 10.1016/j.aca.2021.338842. 34711322

[ref155] Chiavaioli F.; Baldini F.; Tombelli S.; Trono C.; Giannetti A. Biosensing with optical fiber gratings. Nanophotonics 2017, 6 (4), 663–679. 10.1515/nanoph-2016-0178.

[ref156] Al-Nedawi K.; Meehan B.; Rak J. Microvesicles: Messengers and Mediators of Tumor Progression. Cell Cycle 2009, 8 (13), 2014–2018. 10.4161/cc.8.13.8988. 19535896

[ref157] Abels E. R.; Breakefield X. O. Introduction to Extracellular Vesicles: Biogenesis, RNA Cargo Selection, Content, Release, and Uptake. Cell. Mol. Neurobiol. 2016, 36 (3), 301 10.1007/s10571-016-0366-z. 27053351 PMC5546313

[ref158] Zlotogorski-Hurvitz A.; Dayan D.; Chaushu G.; Korvala J.; Salo T.; Sormunen R.; Vered M. Human Saliva-Derived Exosomes: Comparing Methods of Isolation. J. Histochem. Cytochem. 2015, 63 (3), 181–189. 10.1369/0022155414564219. 25473095 PMC4340734

[ref159] Lak N. S. M.; van der Kooi E. J.; Enciso-Martinez A.; Lozano-Andrés E.; Otto C.; Wauben M. H. M.; Tytgat G. A. M. Extracellular Vesicles: A New Source of Biomarkers in Pediatric Solid Tumors? A Systematic Review. Front. Oncol. 2022, 12, 887210 10.3389/fonc.2022.887210. 35686092 PMC9173703

[ref160] Vojtech L.; Woo S.; Hughes S.; Levy C.; Ballweber L.; Sauteraud R. P.; Strobl J.; Westerberg K.; Gottardo R.; Tewari M.; Hladik F. Exosomes in Human Semen Carry a Distinctive Repertoire of Small Non-Coding RNAs with Potential Regulatory Functions. Nucleic Acids Res. 2014, 42 (11), 7290–7304. 10.1093/nar/gku347. 24838567 PMC4066774

[ref161] Jang S. C.; Kim S. R.; Yoon Y. J.; Park K. S.; Kim J. H.; Lee J.; Kim O. Y.; Choi E. J.; Kim D. K.; Choi D. S.; Kim Y. K.; Park J.; Di Vizio D.; Gho Y. S. In Vivo Kinetic Biodistribution of Nano-Sized Outer Membrane Vesicles Derived from Bacteria. Small 2015, 11 (4), 456–461. 10.1002/smll.201401803. 25196673

[ref162] György B.; Szabó T. G.; Pásztói M.; Pál Z.; Misják P.; Aradi B.; László V.; Pállinger É.; Pap E.; Kittel Á.; Nagy G.; Falus A.; Buzás E. I. Membrane Vesicles, Current State-of-the-Art: Emerging Role of Extracellular Vesicles. Cell. Mol. Life Sci. 2011 6816 2011, 68 (16), 2667–2688. 10.1007/s00018-011-0689-3. PMC3142546 21560073

[ref163] Mathivanan S.; Ji H.; Simpson R. J. Exosomes: Extracellular Organelles Important in Intercellular Communication. J. Proteomics 2010, 73 (10), 1907–1920. 10.1016/j.jprot.2010.06.006. 20601276

[ref164] Théry C.; Ostrowski M.; Segura E. Membrane Vesicles as Conveyors of Immune Responses. Nat. Rev. Immunol. 2009 98 2009, 9 (8), 581–593. 10.1038/nri2567. 19498381

[ref165] Skog J.; Würdinger T.; van Rijn S.; Meijer D. H.; Gainche L.; Curry W. T.; Carter B. S.; Krichevsky A. M.; Breakefield X. O. Glioblastoma Microvesicles Transport RNA and Proteins That Promote Tumour Growth and Provide Diagnostic Biomarkers. Nat. Cell Biol. 2008 1012 2008, 10 (12), 1470–1476. 10.1038/ncb1800. PMC3423894 19011622

[ref166] Kwak T. J.; Son T.; Hong J.-S.; Winter U. A.; Jeong M. H.; McLean C.; Weissleder R.; Lee H.; Castro C. M.; Im H. Electrokinetically Enhanced Label-Free Plasmonic Sensing for Rapid Detection of Tumor-Derived Extracellular Vesicles. Biosens. Bioelectron. 2023, 237, 115422 10.1016/j.bios.2023.115422. 37301179 PMC10527155

[ref167] Sun X.; Chen B.; Li Z.; Shan Y.; Jian M.; Meng X.; Wang Z. Accurate Diagnosis of Thyroid Cancer Using a Combination of Surface-Enhanced Raman Spectroscopy of Exosome on MXene-Coated Gold@silver Core@shell Nanoparticle Substrate and Deep Learning. Chem. Eng. J. 2024, 488, 150835 10.1016/j.cej.2024.150835.

[ref168] Feng H.; Min S.; Huang Y.; Gan Z.; Liang C.; Li W.-D.; Chen Y. Concentric Gradient Nanoplasmonic Sensors for Detecting Tumor-Derived Extracellular Vesicles. Sensors Actuators B Chem. 2024, 400, 134899 10.1016/j.snb.2023.134899.

[ref169] Hedhly M.; Wang Y.; Brunel A.; Beffara F.; Akil H.; Verdier M.; Bessette B.; Crunteanu A.; Ho H.-P.; Humbert G.; Lalloue F.; Zeng S. Ultra-Sensitive Real-Time Detection of Cancer-Derived Exosomes Directly from Cell Supernatants by a Large Goos-Hänchen Signal Generation on Plasmonic Sensing Interface. Biosens. Bioelectron. X 2023, 15, 100391 10.1016/j.biosx.2023.100391.

[ref170] Hsu C.-C.; Yang Y.; Kannisto E.; Zeng X.; Yu G.; Patnaik S. K.; Dy G. K.; Reid M. E.; Gan Q.; Wu Y. Simultaneous Detection of Tumor Derived Exosomal Protein-MicroRNA Pairs with an Exo-PROS Biosensor for Cancer Diagnosis. ACS Nano 2023, 17 (9), 8108–8122. 10.1021/acsnano.2c10970. 37129374 PMC10266547

[ref171] Zhu S.; Li H.; Yang M.; Pang S. W. Highly Sensitive Detection of Exosomes by 3D Plasmonic Photonic Crystal Biosensor. Nanoscale 2018, 10 (42), 19927–19936. 10.1039/C8NR07051B. 30346006

[ref172] Fan Y.; Duan X.; Zhao M.; Wei X.; Wu J.; Chen W.; Liu P.; Cheng W.; Cheng Q.; Ding S. High-Sensitive and Multiplex Biosensing Assay of NSCLC-Derived Exosomes via Different Recognition Sites Based on SPRi Array. Biosens. Bioelectron. 2020, 154, 112066 10.1016/j.bios.2020.112066. 32056961

[ref173] Feng J.; Jia L.; Pan W.; Fan Y.; Guo J.; Luo T.; Liu C.; Wang W.; Zheng L.; Li B. Rapid and Efficient Fluorescent Aptasensor for PD-L1 Positive Extracellular Vesicles Isolation and Analysis: EV-ANCHOR. Chem. Eng. J. 2023, 465, 142811 10.1016/j.cej.2023.142811.

[ref174] Jung Y. K.; Son M. H. Polydiacetylene-Based Aptasensors for Rapid and Specific Colorimetric Detection of Malignant Exosomes. Talanta 2024, 268, 125342 10.1016/j.talanta.2023.125342. 37918246

[ref175] Luo Y.; Feng Q.; Ma D.; Wang B.; Chi C.; Ding C.-F.; Yan Y. Highly Sensitive Quantitative Detection of Glycans on Exosomes in Renal Disease Serums Using Fluorescence Signal Amplification Strategies. Talanta 2024, 269, 125467 10.1016/j.talanta.2023.125467. 38042140

[ref176] Liu W.; Li J.; Wu Y.; Xing S.; Lai Y.; Zhang G. Target-Induced Proximity Ligation Triggers Recombinase Polymerase Amplification and Transcription-Mediated Amplification to Detect Tumor-Derived Exosomes in Nasopharyngeal Carcinoma with High Sensitivity. Biosens. Bioelectron. 2018, 102, 204–210. 10.1016/j.bios.2017.11.033. 29145073

[ref177] Yang Y.; Li C.; Shi H.; Chen T.; Wang Z.; Li G. A PH-Responsive Bioassay for Paper-Based Diagnosis of Exosomes via Mussel-Inspired Surface Chemistry. Talanta 2019, 192, 325–330. 10.1016/j.talanta.2018.09.067. 30348398

